# Semi-supervised retrieval of functional residues through the integration of protein language models and gene ontology data

**DOI:** 10.1093/bioinformatics/btag642

**Published:** 2026-08-31

**Authors:** Andrew Dickson, Safaa Mouline, Ali Tamadon, Mohammad R K Mofrad

**Affiliations:** Departments of Bioengineering and Mechanical Engineering, University of California, Berkeley, CA 94720, United States; Departments of Bioengineering and Mechanical Engineering, University of California, Berkeley, CA 94720, United States; Departments of Bioengineering and Mechanical Engineering, University of California, Berkeley, CA 94720, United States; Departments of Bioengineering and Mechanical Engineering, University of California, Berkeley, CA 94720, United States; Molecular Biophysics and Integrative Bioimaging Division, Lawrence Berkeley National Lab, Berkeley, CA 94720, United States

## Abstract

**Motivation:**

Experimental studies of protein function often focus on mechanistic descriptions, characterizing how specific sites and residues contribute to activity. Abstractions such as domains and active sites enable quantitative descriptions of how protein features act biologically. Thanks to the abundance of high-quality sequence and function data, machine learning has achieved great success in directly predicting protein function. However, translating functional characterizations into mechanistic ones on the level of the domains, binding sites, or motifs remains challenging. This represents a semi-supervised problem: sequences and global functional labels are available, but local annotations must be inferred.

**Results:**

We investigate the semi-supervised discovery of functionally active protein regions by integrating protein sequence models with functional information. We first formalize the residue-level functional annotation problem by constructing unified evaluation datasets linking Gene Ontology functions to annotated residues. Eight datasets are assembled, spanning levels of specificity from single active-site residues to domains covering up to 60% of a protein. We then introduce a new class of function-conditioned generative models that more accurately predict functionally important residues than existing approaches, including interpretability methods and PSSM entropy estimation, across multiple benchmark datasets.

**Availability:**

Source code is available at github.com/mofradlab/go_interp, with an archived snapshot at doi.org/10.5281/zenodo.21401001.

## 1 Introduction

### 1.1 Problem and gap

While protein machine learning models achieve high performance on functional classification tasks (Boadu *et al.* 2025), it is difficult to directly characterize the elements of a protein responsible for their function. Often experimental data comes in the form of high-level information on function, such as knock-out experiments, or the results of high-throughput experiments, in which sequence level information on protein function is available, but residue level annotations may be far more difficult to acquire.

It is common to build databases of conserved regions, motifs, or sites, and functionally annotate them through comparison to real world experiment, such as in the Conserved Domain Database (CDD) (Marchler-Bauer *et al.* 2011), or the PRINTS database of protein fingerprints (Attwood *et al.* 2003).

### 1.2 Prior methods and limitations

While there are dozens of existing methods for predicting conserved elements in proteins, most rely purely on sequence or evolutionary information. Prior methods typically rely on global importance estimates, in which the general relevance of residues are estimated through methods such as sequence conservation, and these are used for a proxy for functional importance. Sequence-based methods, such as ConSurf (Ashkenazy *et al.* 2016), SIFT (Sim *et al.* 2012), and those using information-theoretic measures like Jensen-Shannon Divergence (JSD) (Capra and Singh 2007), typically estimate a position-specific scoring matrix (PSSM) for residues using multiple sequence alignments of homologous sequences. Common pipelines include a homology search, such as through PSI-BLAST, followed by multiple sequence alignment through tools such as MAFFT or MUSCLE (Altschul *et al.* 1997, Edgar 2004, Katoh and Standley 2013). At the same time, modern pre-trained generative models over protein sequences, like those from the ESM (Evolutionary Scale Modeling) family (Lin *et al.* 2023), may be used to estimate the same scoring statistics directly without needing an explicit alignment (Meier *et al.* 2021).

However, because these methods ignore experimental functional information they cannot distinguish conservation for functional reasons from conservation for reasons such as structural stability or alternative functions (Pancsa and Fuxreiter 2012). Annotated datasets, such as the CDD or PRINTS, or larger aggregates such as InterPro (Hunter *et al.* 2009), typically rely on post-hoc annotation of identified regions, instead of directly integrating functional information into sequence annotations. There is a need for methods capable of accounting for prior knowledge of protein function, but the effectiveness, and feasibility, of potential approaches are unclear.

### 1.3 Contributions

In this work, we formalize semi-supervised functional residue prediction by constructing a benchmark of eight datasets. We then systematically compare the retrieval performance of conservation-based methods, classifier interpretability techniques, and generative language models. Finally, we introduce a novel function-conditioned generative model trained with a context-span masking objective for the purpose of incentivizing the use of functional information.

A natural way to predict functional information is to extract residue level predictions from function classifier models, such as predictors of Gene Ontology (GO) annotations (Ashburner *et al.* 2000, Camon *et al.* 2004). Existing machine learning models trained on annotations already outperform sequence search based methods (Sanderson *et al.* 2023), and in particular, pre-trained protein language models (pLMs) fine-tuned on annotation data show great success in producing high-quality annotations (Littmann *et al.* 2021); for a comprehensive survey of deep-learning methods for protein function prediction, see Wang *et al.* (2025). We apply multiple interpretability algorithms to a strong pLM for a residue retrieval task. At the same time, we compare against several sequence conservation-based approaches using MSAs or generative models.

We find that in most cases a generative modeling, entropy-based, approach is stronger than extracting residue-level information from even a strong classifier. Naive training for classification appears to lose or ignore information relevant to the sequence distribution, losing important residue level information. Notably, we do find that in cases of high-entropy, high-relevance residues such as those in the Eukaryotic Linear Motif resource (Kumar *et al.* 2019), interpretability based methods show the strongest overall performance.

For most problem settings, we find that functional information, in the form of global assignment of Gene Ontology terms to protein sequences, can be integrated into predictions of residue importance through the correctly incentivized fine-tuning of generative sequence models. For the best overall retrieval of function-relevant residues with an semi-supervised method, we develop a protocol for fine-tuning pre-trained models to solve an artificially challenging masked language modeling objective conditional on functional information.

## 2 Materials and methods

### 2.1 Benchmark datasets

For our evaluations, we build eight datasets with residue-level annotations Fig. 1. Two datasets identify active sites from the Catalytic Site Atlas and InterPro, and two identify binding sites from BioLiP2 and InterPro (Hunter *et al.* 2009, Ribeiro *et al.* 2018, Zhang *et al.* 2024). We also include a dataset of short linear motifs from the Eukaryotic Linear Motif resource, a dataset of regions driving liquid-liquid phase separation (LLPS) from PhaSePro, and finally, two datasets of functionally implicated repeats and domains, also extracted from InterPro (Kumar *et al.* 2019, Mészáros *et al.* 2020). InterPro is an aggregate of annotated regions from over a dozen databases, which we broadly decompose into four classes of entries: those representing active sites, binding sites, repeat regions, and domains.

**Figure 1:**
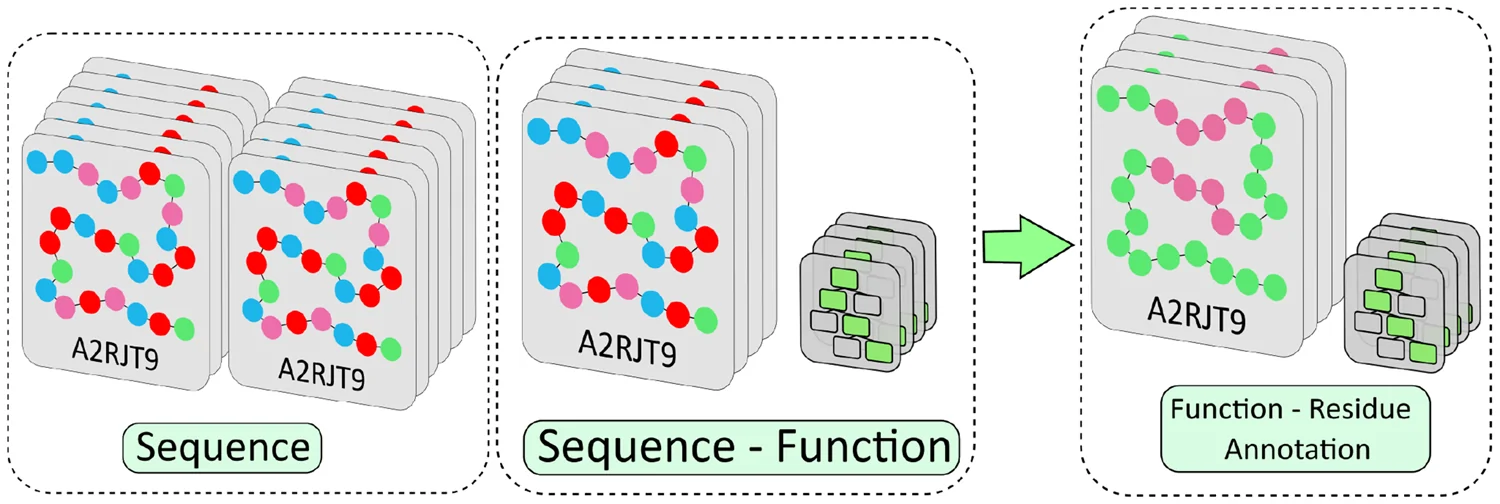
Dataset structure for the residue level functional annotation problem. The depicted protein sequence, and sequence-function datasets may be constructed from large existing databases. From these, we construct and evaluate position-level models designed to recover predictions of functionally active residues, indicated on the right.

For the CSA, catalytic-residue indices are extracted directly from the M-CSA flat-file export, and EC numbers are converted to Gene Ontology terms via the EC2GO mapping file (Tipton 1992, Ashburner *et al.* 2000). For the ELM dataset, motif instances are merged with the GO-term table on ELM identifier; records are aggregated per UniProt ID and ELM class so that each ELM class contributes at most one entry per protein, yielding 304 entries. For the LLPS dataset, phase-separating region boundaries are parsed from PhasePro’s semicolon-delimited start–end ranges, and GO terms are assigned by matching organelle annotations in PhasePro against GO term names using the Gene Ontology basic ontology file. Entries are retained only when the total annotated region length is less than 75% of the full sequence, excluding proteins whose entire sequence constitutes the functional annotation. For BioLiP, binding-site residue indices are extracted from the binding-site residue field of the non-redundant BioLiP dataset. For InterPro-derived datasets, residue-level annotations are taken from the domain boundary coordinates in the protein2ipr mapping file, and InterPro entries are pre-filtered to those with more than 10 000 member proteins; we then build each of our four InterPro datasets by downsampling to a small set of representative region identifiers. For full details on dataset construction, see the Supplementary Materials S1.

Each dataset is filtered to proteins of length of at most 850 residues containing regions associated with at least one gene ontology term.

Because of their large size, and to produce datasets of comparable sizes to the CSA, ELMS, and LLPS datasets, we downsample the BioLip and InterPro derived datasets to between 100 and 1000 proteins (Table 1). In the case of BioLiP, we select out the top 12 most common binding ligands and sample 50 proteins for each. For each selected InterPro category, we sample 12 representative identifiers, and for each identifier sample 50 associated proteins. We then filter out domains or regions covering over 60% of a protein sequence, and annotate regions using the interpro2go database (Hunter *et al.* 2009).

**Table 1:** Summary statistics of residue annotation datasets.

Dataset	Residues	Annotations	Perc. coverage
CSA	$3.00\times 10^{5}$	$3.59\times 10^{3}$	0.01
IP Active Site	$1.67\times 10^{5}$	$5.56\times 10^{3}$	0.04
IP Binding Site	$1.87\times 10^{5}$	$8.51\times 10^{3}$	0.06
ELMS	$1.06\times 10^{5}$	$2.16\times 10^{3}$	0.03
BioLip	$2.50\times 10^{5}$	$5.51\times 10^{3}$	0.04
LLPS	$2.98\times 10^{4}$	$1.10\times 10^{4}$	0.37
IP Repeat	$1.45\times 10^{5}$	$1.73\times 10^{4}$	0.15
IP Domain	$8.76\times 10^{4}$	$2.96\times 10^{4}$	0.33

### 2.2 Sequence resources and GO annotations

We construct a dataset of high quality protein sequences and functional annotations using the SwissProt (Camon *et al.* 2004), Gene Ontology Annotation database, and Gene Ontology datasets using our previously developed gobench.org web application (Dickson *et al.* 2023). We filter for annotations sourced from human-reviewed experimental codes (Tipton 1992) selected and apply otherwise default settings. In total, the dataset contains 1 12 000 proteins, with an average of ten annotations for each protein. We apply an 80–20 train-validation split; the validation set is used solely to monitor masked language modeling loss and is not used for residue-level evaluation. We did not exclude benchmark proteins from the SwissProt-GOA fine-tuning dataset, but no residue-level annotations are used at any point during training. All residue-level evaluation is performed exclusively on the eight benchmark datasets described in Section 2.1 (CSA, IP Active Site, IP Binding Site, ELMs, BioLiP, LLPS, IP Repeat, IP Domain), which are curated from entirely independent databases and were never seen during training; these constitute the held-out test data for the residue-level attribution task. At all stages of evaluation, we assume that both sequence $S_{i}$ and a set of protein-level GO labels $G_{i}$ are available as inputs, and we then seek to infer residue-level importance scores conditioned on $\left(S_{i},G_{i}\right)$, rather than to evaluate generalization of GO prediction itself.

### 2.3 PSSM construction

To construct Position-Specific Scoring Matrices (PSSMs), we first searched the UniRef90 database (Suzek *et al.* 2015) using MMSeqs2 (Steinegger and Söding 2017). After filtering homologous sequences based on identity and length, we generated Multiple Sequence Alignments (MSAs) with Muscle5 (Edgar 2022). From the MSA, PSSM scores were calculated from amino acid counts at each position, with residue columns having ≥30% gaps assigned maximum entropy, following (Capra and Singh 2007). For full details, see the Supplementary Materials S3.

### 2.4 Protein language models and training

For classifier interpretability and generative modeling methods for predicting functional importance, we use the pre-trained protein language model, ESM-Cambrian (ESM-C), which we selected for its state-of-the-art masked language modeling performance and efficient inference among openly available pLMs, though our framework is model-agnostic and applies to any masked pLM. For function prediction, we fine-tune the model on Gene Ontology (GO) term classification, achieving a maximum F1-score of 0.83 on macro-averaged GO terms in our validation set, comparable with strong models from prior studies (Dickson *et al.* 2023). For generative modeling, we introduce a method to condition the ESM-C model on GO terms to generate protein sequences (Fig. 2). We find that a novel context-span masking objective is necessary for the model to most exploit functional information during generation.

**Figure 2:**
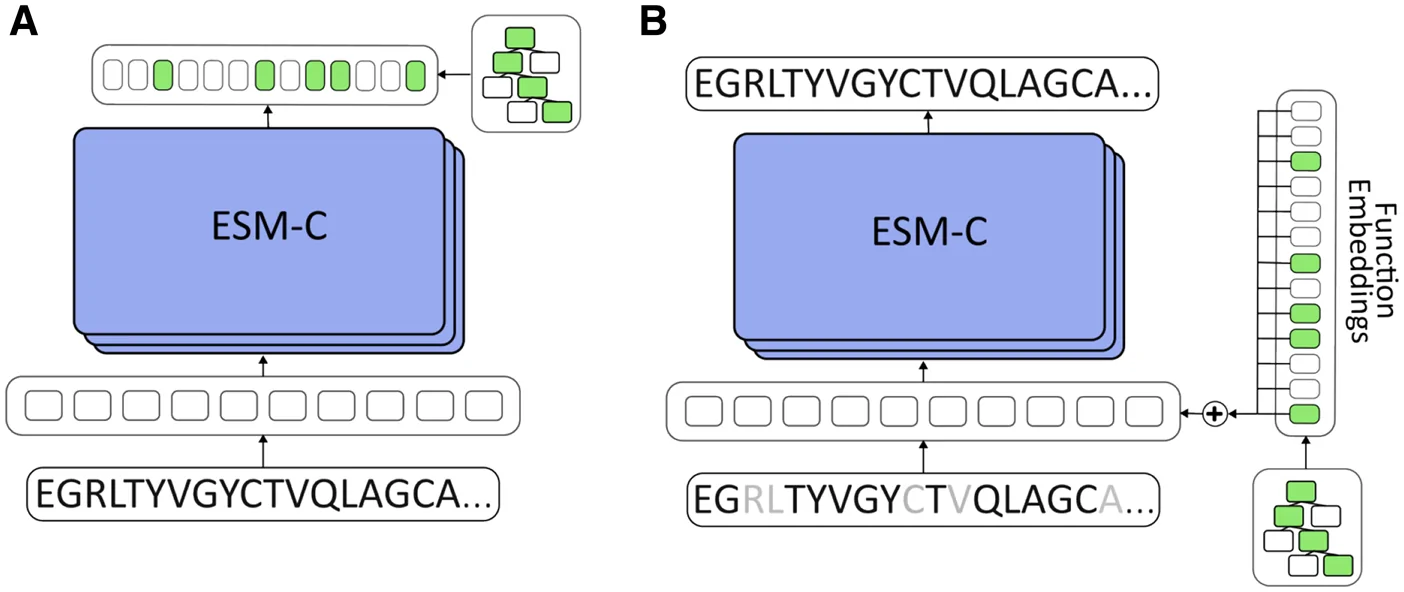
(A) The fine-tuning architecture for a GO classifier model. We replace the token logit output of the pre-trained sequence model with a biased linear transform of the average of all residue embeddings from the final layer of ESM-C. Outputs are treated as logits for each Gene Ontology class, and trained with binary cross entropy. (B) The fine-tuning architecture for a function conditioned masked modeling model over protein sequences. While the pre-trained ESM-C output is left unchanged, the input residue sequences are perturbed by a sequence level functional embedding.

Our work uses a dataset D of *N* protein sequences, $D={\left\{(S_{i},G_{i})\right\}}_{i=1}^{N}$. Each protein sequence $S_{i}$ is a series of amino acid residues, $S_{i}=(r_{1},r_{2},\ldots ,r_{L})$, where *L* is the sequence length. The function of each protein is described by a set of Gene Ontology (GO) terms $G_{i}\subseteq G$, where G is the complete set of GO terms. For classification, we represent $G_{i}$ as a multi-hot binary vector $y_{i}\in {\left\{0,1\right\}}^{\left|G\right|}$.

The primary model used for fine-tuning and generative modeling was the 600M ESM-Cambrian protein language model, trained on a masked language modeling objective derived from the original BERT training procedure (Devlin *et al.* 2019, Lin *et al.* 2023, Hayes *et al.* 2024). For managing sequence data, we use the BioPython package (Cock *et al.* 2009). For machine learning training and inference we use the PyTorch and PyTorch Lightning packages (Falcon and The PyTorch Lightning Team 2019, Paszke *et al.* 2019). For evaluation we use the Pandas, NumPy, and Scikit-learn packages (Pedregosa *et al.* 2011, Harris *et al.* 2020, The pandas Development Team 2020).

We adopt a two-phase fine-tuning strategy for our GO classification problem setting. Let the ESM-C encoder be denoted by $f_{\theta }$, where θ represents the model parameters. For a given sequence *S*, the model produces final-layer residue embeddings $H=(h_{1},h_{2},\ldots ,h_{L})=f_{\theta }(S)$, where $h_{i}\in R^{d}$.

These embeddings are averaged to produce a sequence-level representation, which is passed to a linear classification head. The final probabilities $\hat{y}$ are obtained by applying an element-wise sigmoid function, $\hat{y}=\sigma (z)$ (Fig. 2).

The classification model is trained by minimizing the binary cross-entropy (BCE) loss over all GO annotations:


$$L_{\mathrm{BCE}}(y,\hat{y})=-\sum_{j=1}^{\left|G\right|}\left[y_{j}\;\text{log}({\hat{y}}_{j})+(1-y_{j})\;\text{log}(1-{\hat{y}}_{j})\right]$$

Because each protein may be annotated with multiple GO terms, GO assignment is a multi-label rather than a multiclass problem, for which per-term binary cross-entropy with sigmoid outputs is the standard loss. The first fine-tuning phase trains only the classification head (*W*, *b*) with the ESM-C parameters *θ* frozen, using a high learning rate. Following prior methodology (Dickson *et al.* 2023), the second phase fine-tunes the entire model (*θ, W, b*), truncated at five epochs with early stopping, with a low learning rate annealed from $1\times 10^{-5}$ to $1\times 10^{-6}$. Our final classifier achieves a maximum F1-score of 0.83 on our validation data, averaged over all GO classes.

### 2.5 Function conditioned fine-tuning

Given our dataset of paired protein sequences and GO terms, we also fine-tune the generative sequence model to condition on functional information. We augment the pre-trained ESM-C model with a learnable GO term embedding matrix $E_{GO}\in R^{|G|\times d_{\mathrm{emb}}}$.

During evaluation of a protein with associated GO terms *y*, the corresponding embedding vectors are summed to create an aggregate function vector $v_{\mathrm{func}}=\sum_{j=1}^{\left|G\right|}y_{j}E_{GO}^{\left(j\right)}$.

A linear transform of this aggregate vector is then added to each initial residue embedding $x_{i}$ at the first layer of the ESM-C transformer to perturb the model’s output: $x_{i}^{\prime }=x_{i}+(W_{p}v_{\mathrm{func}}+b_{p})$ where $W_{p}$ and $b_{p}$ are learnable projection parameters. The ESM-C and function embedding parameters are then jointly fine-tuned on a masking loss objective.

We then fine-tune the function-conditioned ESM-C model on the standard masked modeling task, where a set of random indices $M_{\mathrm{rand}}$ corresponding to 15% of the sequence residues are masked. The loss is then.


$$L_{\mathrm{MLM}}=-\sum_{i\in M_{\mathrm{rand}}}\;\text{log}\;P(r_{i}|\tilde{S},y)$$

### 2.6 Context span masking objective

During evaluation, we find that function conditioned fine-tuning on a BERT-style random masking objective produces results almost identical to baseline, indicating that functional information is not sufficiently utilized by our model under the default training objective (Section 3.3). We hypothesized that the standard masked language modeling objective is too simple to force the model to account for global functional information.

Masking contiguous spans is known to increase the difficulty, and potentially the quality, of masked language modeling (Joshi *et al.* 2020), while protein models are known to rely in part on full-sequence context to infer domain distributions (Zhang *et al.* 2024). Based on both results, we developed a more challenging training objective, termed context-span masking, designed to obscure larger, contiguous blocks of local information, and remove all knowledge of the protein sequence outside of a small context window.

In this modified task, models are forced to focus on contiguous subsequences. For a sequence of length *L*, we define a random context region of length one hundred within the full protein sequence. Residues outside this region are masked and excluded from the loss calculation. Within the context region, residues are masked in contiguous spans until 30% of the total residues in the context window are masked, and the model is trained on the same cross-entropy loss. We evaluate context lengths of 50, 100, and 200 residues, and span masking lengths of 2, 5, and 10, as fine-tuning objectives and adopt a span-masking length of 5 in a context region of 100. For full benchmarking of parameters, see Table S3.

For both fine-tuning processes, the pre-trained model is fine-tuned with functional conditioning for up to five epochs at a learning rate of $1\times 10^{-5}$ annealed to $1\times 10^{-6}$. Training employs early stopping on the validation masked language modeling loss with a patience of two epochs, with testing indicating best performance is reached in early epochs (Tables S3 and S4). As a baseline, we also fine-tune the base ESM-C model on the same masking objectives and dataset without functional conditioning.

### 2.7 Estimating entropy and entropy-gain

PLM predictions are known to estimate the likelihoods of potential residue mutations (Meier *et al.* 2021). To estimate a position specific scoring matrix from a masked language model, we average predictions of the categorical distribution of each residue (Hayes *et al.* 2024, Zhang *et al.* 2024). A large batch of masked sequences is generated from a target sequence such that each residue is masked at least once within the batch. We then estimate the categorical distribution of amino acids at each masked position with application of our model and average over all predicted distributions for each position to produce a PSSM estimate.


$$\hat{R_{i}}=\frac{1}{N_{\mathrm{cov}}}\sum_{\tilde{S}\in \mathrm{Batch}}f_{\theta }(r_{i}|\tilde{S})\cdot I({\tilde{S}}_{i}=t_{\mathrm{mask}})$$

We may then estimate a positional entropy $h_{\theta }(R_{i})=-\sum_{r_{i}}P(R_{i}=r_{i})\cdot \log[P(R_{i}=r_{i})]$.

For the base ESM-C model we randomly mask batch elements until fifteen percent of the total are overwritten, and generate batches of masked sequences large enough that the amino acid distribution for each residue is predicted at least six times in total. Based on calibration tests on the CSA dataset, we find that for pre-trained models retrieval performance improves with the number of masked predictions for each residue, but that performance saturates quickly and is largely robust to masking strategy (see Tables S1 and S2).

For models fine-tuned on the context-span masking objective, we generate context-span formatted masks for batches, and average only over masked tokens within the open window region, to match the fine-tuning training objective.

To isolate the effect of function conditioning in masked language modeling, we take the additional step of calculating entropy gain scores for our fine-tuned generative models. Here, entropy estimates from function-conditioned models are subtracted from the entropy estimates of baseline models fine-tuned on the same data without access to functional information (Section 3.3). The resulting scoring function is $g_{\theta ,\theta_{\mathrm{baseline}}}(R_{i})=h_{\theta_{\mathrm{baseline}}}(R_{i})-h_{\theta }(R_{i})$, which corresponds to the reduction in entropy of a residue’s distribution from the inclusion of functional information (Fig. 5).

### 2.8 Interpretability methods

Given a strong classifier, interpretability methods provide a method for predicting the importance of features to the model’s output, implicitly predicting the importance of residue features to GO functions.

To attribute classifier predictions to specific residues, we employed four methods from the Captum package (Kokhlikyan *et al.* 2020): Layer-wise Integrated Gradients (Sundararajan *et al.* 2017), Gradient X Activation (Sundararajan *et al.* 2017), DeepLIFT (Shrikumar *et al.* 2019), and GradientShap (Lundberg and Lee 2017). For each method, we used the L2 norm of the attribution scores over the residue embedding dimensions to produce a single importance score per residue.

We also include a simple attention-weight baseline based on prior work on protein interpretation (Nayar *et al.* 2025). For each protein, we extract self-attention maps from the final layer of the GO classifier, average attention weights over all heads, and then average incoming attention for each residue position. The resulting scalar per-residue attention scores are used as an alternative, non-specific, estimate of importance.

### 2.9 Evaluation metrics

Scoring methods are evaluated either by the rank they assign functionally annotated residues or by their ability to positively classify functionally annotated residues.

We calculate each rank-based method on a per-protein basis, and then average over proteins for a dataset level statistic. Scores over protein residues are converted to importance rankings.

To evaluate the number of retrievals required to find a first relevant residue, we calculate the mean reciprocal rank (MRR) (Radev *et al*. n.d). For a given protein, the reciprocal rank corresponds to the inverse of the lowest rank of a relevant residue. We average over all proteins to calculate MRR. We apply MRR in particular to the case in which a small percentage of protein residues are relevant, such as for datasets of active or binding sites.

To evaluate the percentage of relevant residues in a small initial set of retrievals, we also calculate the fraction of the top thirty ranked residues which are in the set of functionally relevant positions (Capra and Singh 2007). For proteins with less than thirty relevant residues, the top-30 metric is reweighted so that placing all relevant results within the first thirty residues will receive a perfect score of 1.0. Top-30 percentages are averaged over all proteins for the final dataset metric.

For meanAUC calculation, we adopt methods previously applied to sequence conservation estimates (Capra and Singh 2007), in which the area under the reciever–opererator characteristic curve (ROC-AUC) is calculated for reach protein, with functionally-annotated residues as positive datapoints and the remainder as negative. AUC scores are then averaged to produce the meanAUC for a dataset.

## 3 Results

### 3.1 Masked modeling entropy estimates outperform MSA

Both multiple sequence alignment and generative masking methods estimate the position specific distribution over residues for a protein. As an initial baseline, we compared their performance across the CSA, ELMS, LLPS, and InterPro domain datasets, selected for their diversity in specificity and function. Both MSA and masked modeling based PSSMs are converted into positional importance values by calculating the entropy of the categorical distribution over possible amino acids.

While both estimates produce similar results on annotation retrieval across datasets, ESM-C demasking predictions outperform MSA across the board, with between a 2% and 5% increase in meanAUC performance for most datasets, and a comparable increase across almost every metric (Table 2). Notably, both methods are worse than random for the case of retrieving important disordered domains, although demasking does achieve considerably higher performance overall. Because of the dominance of masked modeling for residue retrieval across datasets, and the high computational cost of constructing complete MSAs, we restrict our focus to masked modeling for following benchmarks.

**Table 2:** Comparison of PSSM and demasking entropy for annotation retrieval.

Dataset	MSA			ESM-C		
	AUC	MRR	Top-30	AUC	MRR	Top-30
CSA	0.79	0.19	0.42	**0.82**	**0.40**	**0.59**
ELMS	0.50	0.05	**0.09**	**0.52**	**0.077**	**0.09**
LLPS	0.38	0.30	0.24	**0.45**	**0.33**	**0.30**
IP Domain	0.74	0.62	0.61	**0.79**	**0.79**	**0.70**

Performance was evaluated across four datasets using Area Under the ROC Curve (AUC), Mean Reciprocal Rank (MRR), and recall for the top 30 predictions (Top-30), highest performing entries indicated in bold. ESM-C demasking outperforms PSSM across almost all metrics and datasets.

### 3.2 Benchmarking of classifier interpretability methods

To determine the relative effectiveness of interpretability methods, we apply the gradient × activation, layer deep lift, integrated gradients, and SHAP methods to each of our assembled datasets of proteins (Table 3). The annotated region of a protein may be associated with multiple GO terms, in which case we select the deepest term from the GO-DAG for evaluation, on the basis that this carries the most specific information on the region’s function. Aggregating attributions across all positive GO terms instead of this single term yields only marginal and inconsistent changes in retrieval performance (Supplementary Section S5), so we retain the most-specific term throughout.

**Table 3:** meanAUC scores for each interpretability method on all datasets.

Dataset	**LGA Grad** × **Act.**	LDL DeepLIFT	LGS GradSHAP	LIG Integ. Grad.	Naive
CSA	0.60	**0.62**	0.54	0.54	0.50
IP Active	**0.58**	0.57	0.49	0.48	
BioLip	0.60	**0.62**	0.57	0.57	
IP Binding	**0.59**	0.58	0.51	0.50	
ELMS	**0.59**	**0.59**	0.55	0.56	
LLPS	0.49	0.49	0.50	**0.51**	
IP Repeat	0.47	0.48	0.51	**0.53**	
IP Domain	**0.55**	**0.55**	0.46	0.50	

meanAUC is the unweighted average of AUC scores over all proteins in a dataset, highest performing entries indicated with bold. In all cases the naive performance is meanAUC of 0.5.

While all interpretability methods achieve positive signal on most datasets, they provide largely non-specific results, with LDL and LGA achieving meanAUC on the order of 0.6 for most datasets (Fig. 3). Qualitatively, readouts of interpretability scores show very noisy relevance predictions, with a slight bias towards positively annotated sites (Fig. 3B). Interpretability methods do show qualitatively similar prediction patterns, and output highly correlated residue rankings (Fig. 3B), indicating extraction of similar data from the base classifier.

**Figure 3:**
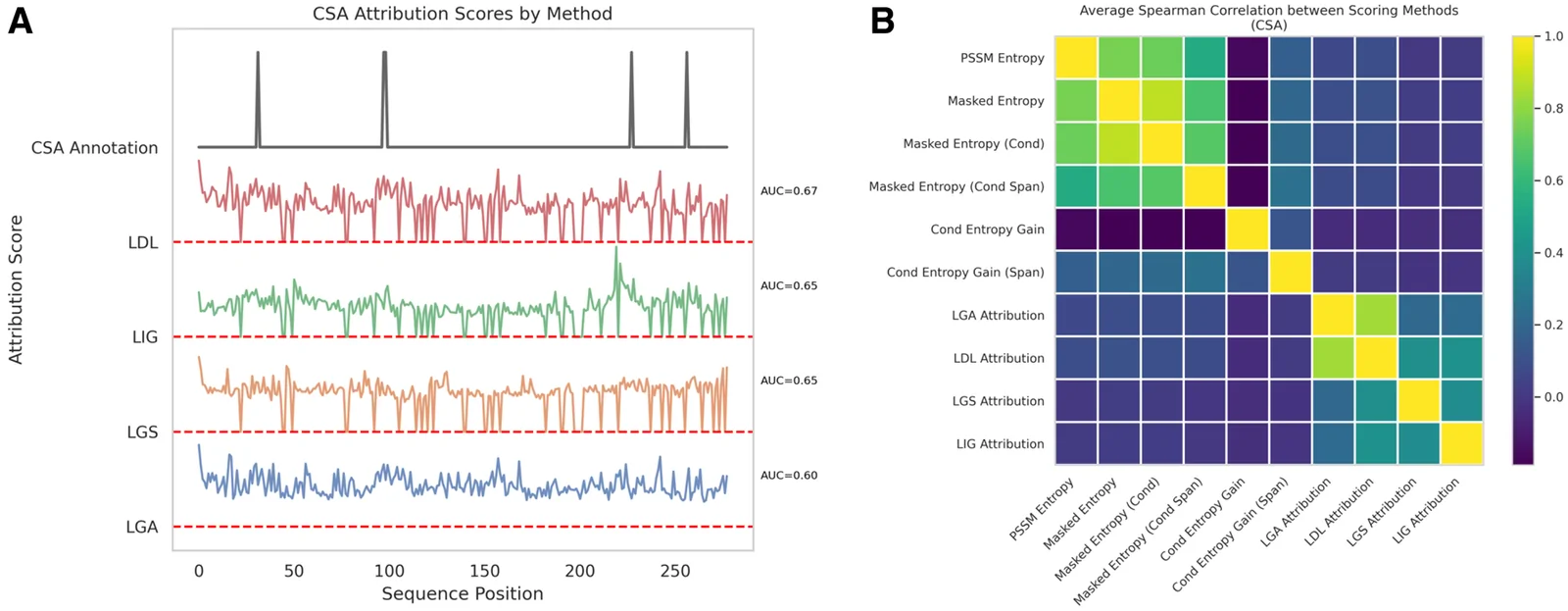
(A) Case study of interpretability methods predicting residue importances. Layer attribution methods applied to UniProt O31168 protein to estimate contribution of residues to a positive prediction of chloride peroxidase activity, plotted as predicted importance score against protein residue index. (B) Averaged Spearman’s rho coefficients of residue rankings from different methods on the CSA dataset, depicted as a heatmap of pairwise coefficients.

**Figure 4:**
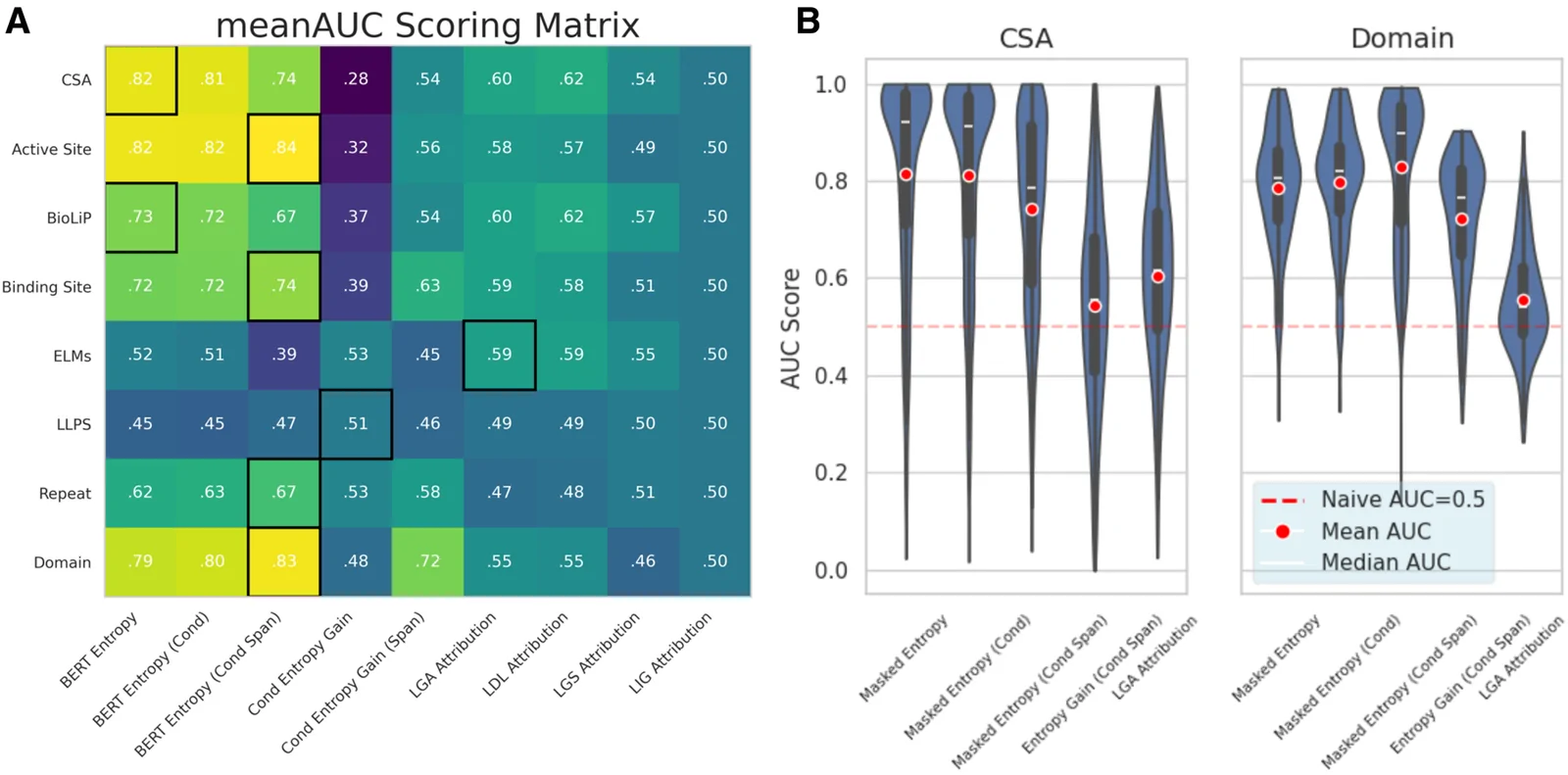
(A) All-dataset comparison of Masked Entropy, Function-Conditioned Entropy, and classifier and attention interpretability methods on retrieval of functionally important residues, as evaluated by meanAUC, depicted as a table of dataset plotted against attribution method score. Highest performing model outlined in black for each dataset. (B) Violin-plots of the distribution of per-protein AUC scores. Mean and median meanAUC plotted for each model in red dot and white dash respectively.

### 3.3 Retrieval comparison on all datasets

In our final comparison, we evaluate both ESM-C derived entropy estimation methods and classifier interpretability methods on each of our constructed datasets in Fig. 4. We additionally quantify residue importance by the sum of in-going head attention values across all residues.

Because of its applicability to all datasets, at all annotation specificity levels, we use meanAUC scores for comparison between methods. While Top30 metrics show similar results, MRR metrics tend to favor demasking entropy models with higher performance on outlier residues such as active site amino acids, as shown in Fig. S1. Because the GO term is a conditioning input to our retrieval task rather than a prediction target, performance is not systematically confounded by GO-term depth: stratifying each dataset at its median depth changes meanAUC for the entropy models by less than 0.013 on average, with a dataset-dependent sign (Supplementary Section S6).

#### 3.3.1 Naive entropy methods dominate for highly specific retrieval

During our full comparison, we find that for both the CSA and BioLiP datasets it is most effective to score residues by entropy estimates, without regard for function (Section 3.3). We hypothesize that for extremely important residues within a protein, such as active sites or critical residues within binding sites, conservation is sufficiently high that functional information is redundant. In these cases, direct masked entropy estimates dominate, achieving meanAUC of 0.82 and above on the CSA dataset (Table 2). However, they show much poorer performance for potentially low entropy regions, in particular for repeat and domain regions extracted from the InterPro dataset (Section 3.3).

#### 3.3.2 Functional information improves retrieval for functional regions

In most cases, function conditioned entropy performs similarly to entropy derived from our pre-trained model. We hypothesize that under the default 15% masking scheme used for residue distribution estimation functional information is largely redundant. This is supported by the performance of conditional entropy gain as a retrieval metric, in which gain under default masking correlates negatively with entropy-based predictions (Fig. 3), and worse than random on most tasks.

With the introduction of context-span masking, we find that our model gains significantly more information from protein function. The span-masked model achieves the highest performance out of all models on all InterPro derived datasets (Section 3.3). In addition, entropy-gain derived from the span-masked and span-mask baseline models achieves significant positive signal on all but the ELMS and LLPS datasets, and outperforms baseline interpretability methods (Section 3.3).

Qualitatively, such as in Fig. 5, we see that the most significant areas of entropy gain are in functionally relevant regions. Entropy-gain may be thought of as an additional, functionally determined, signal, which may be added to the baseline entropy prediction to produce a more discriminative prediction.

**Figure 5:**
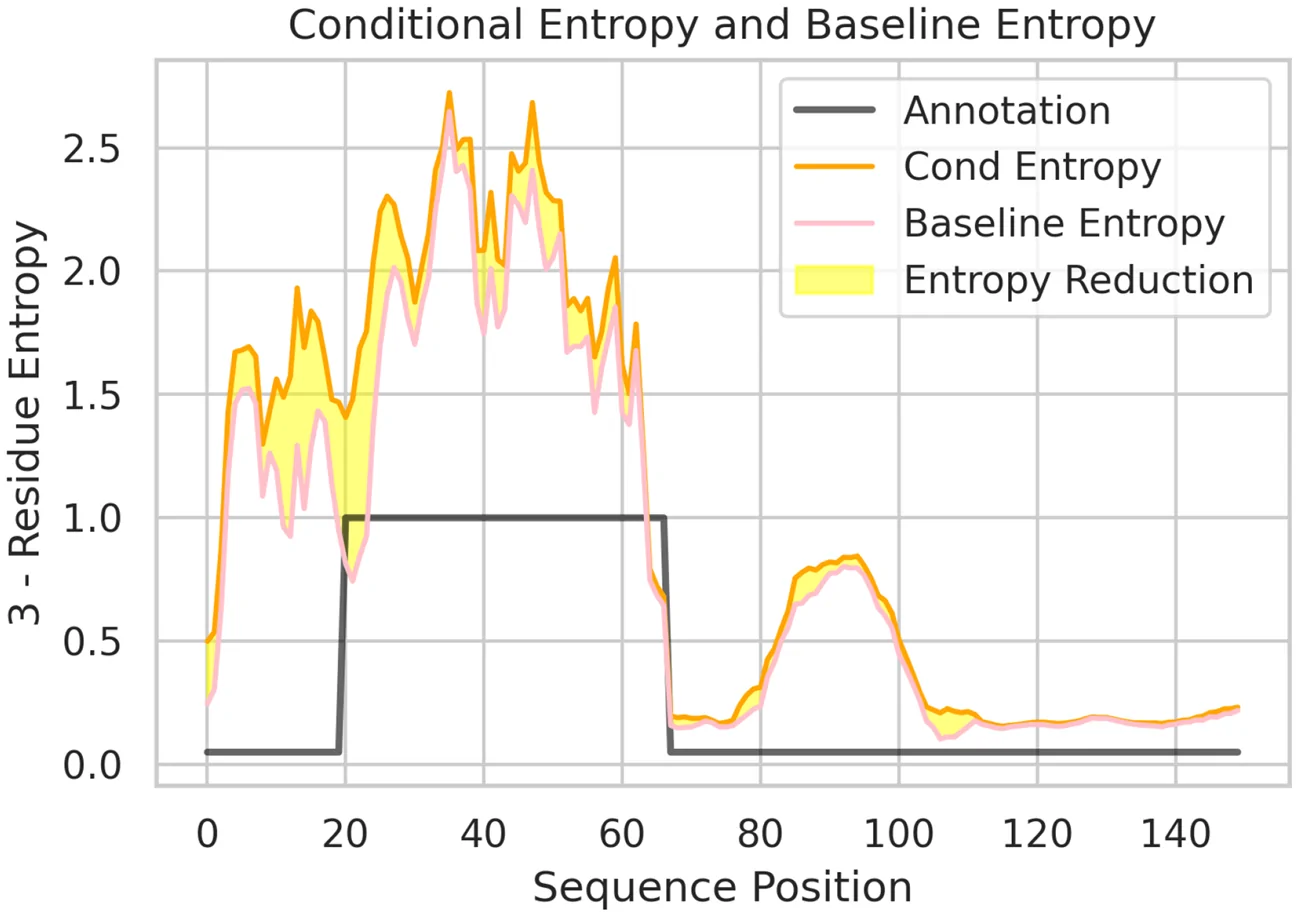
Case study of the effect of functional information on residue entropy. A $3-h_{\theta }(r_{i})$ score plotted for both function conditioned and baseline residue entropy estimates. Functional information lowers model entropy on function relevant regions, increasing retrieval rate.

One of the costs of the span-masking objective is diminished scoring on the highest specificity datasets such as CSA. Missing information, and missing local context from the masking pattern, increases overall model uncertainty on highly specific residues.

In almost all cases, interpretability-based methods derived from a classifier perform poorly for residue retrieval Table 3. The exception is for the ELMS dataset, in which layer gradient activation and layer deep lift both achieve significant signal, and convincingly outperform all other methods. Notably, the ELMS dataset is composed of relatively high entropy residues, which may not correspond to truly low entropy regions even for a perfect model with access to functional information. This may represent an adversarial example for direct sequence conservation-based methods. While attention-based methods do tend to outperform other forms of interpretability, we do not find they are competitive with entropy-based methods for any problem setting.

We also find that all datasets perform with close to random performance on the LLPS dataset, in which disordered regions represent a challenging target for both classifiers and generative models. Because the LLPS GO labels are derived from a manual mapping of free-text condensate descriptions to GO terms, they are a noisier and more subjective proxy than our other benchmarks, so this near-random performance likely reflects both the intrinsic difficulty of disordered, low-complexity regions and weaker annotation quality. High performance on the LLPS dataset may require explicit treatment of disordered regions, or more powerful models of the sequence distribution.

## 4 Discussion

Determining the functional importance of protein residues is a critical, but at the same time fundamentally ill-posed, problem. Proteins may have multiple functions (Jeffery 2018), critical structural elements (Ismi *et al.* 2022), or dozens of other conserved properties that may be so entangled with function that they are inseparable.

In agreement with previous research into sequence conservation (Capra and Singh 2007), we find that residue entropy derived from PSSM estimates shows extremely high correlation with residue functional relevance, especially in the case of the most highly conserved locations such as catalytic sites. On other datasets, with the notable exception of ELMS motif sequences, they show similarly strong performance, despite using no functional information whatsoever in their predictions.

Structure-aware function predictors, such as DeepFRI (Gligorijević *et al.* 2021), score residues by combining sequence with experimentally determined or predicted structure. Where high-quality structures are available, such methods carry a higher ceiling for spatially defined features such as catalytic and binding sites, whose residues are often contiguous in three dimensions while distant in sequence. This dependence on structural input is also their central limitation: coverage is constrained for proteins lacking experimental structures or with low-confidence predicted models, and for the intrinsically disordered regions—such as those driving phase separation—where our results show all sequence-based methods already struggle and for which reliable structures do not exist by definition. Our setting is deliberately structure-free, requiring only sequence and protein-level GO labels, and is therefore applicable uniformly across the proteome, including the regions least amenable to structural modeling. We thus regard sequence-based and structure-aware residue scoring as complementary rather than competing, with the integration of structural information into the context-span masking and entropy-gain framework a natural extension of this work.

Our results indicate that the greater availability, and higher information density, of sequence datasets allows sequence based models to achieve high performance on residue retrieval tasks. The high performance of MLM entropy supports a long-running trend in bioinformatics research (Zuckerkandl and Pauling 1965, Capra and Singh 2007, Ashkenazy *et al.* 2010), in which residue entropy is a heuristic indicator for functional relevance. Across all datasets and metrics, we observe better retrieval from MLM sequence models than MSA alignments, with the ESM-C model showing better generalization than direct alignment of homologous sequences. The high correlation of ESM-C and MSA estimates of residue distributions indicates that both are estimating similar protein sequence properties from similar data, but that the higher flexibility of direct generative modeling eventually produces stronger results.

In the case of regions which may not be uniformly conserved such as domains, repeat regions, or less specific active or binding sites, functional information becomes more important for specific retrieval. For these cases, we demonstrate that knowledge of a protein’s intended function can augment generative models. While function information shows little benefit for models trained on a typical 15% masking objective, it significantly influences models trained on the more challenging context-span masking objective. We confirm that these function-conditioned models have the highest retrieval performance for broader annotation types, and through a comparison to baseline models we verify that the reduction in residue entropy from this functional knowledge constitutes a signal for relevance, i.e. independent of sequence conservation.

A critical limitation for all methods is their failure in cases of high sequence disorder, as is the case for ELMS motifs and intrinsically disordered domains from the LLPS dataset. Although these regions are experimentally confirmed to be critical for protein function (Kumar *et al.* 2019, Mészáros *et al.* 2020), they do not show significantly low entropy. All methods perform comparably to random chance on the LLPS dataset, while only interpretability methods achieve significant signal on the ELMS dataset.

A fully-generative model, trained from scratch to reconstruct function and sequence simultaneously, would likely show stronger performance in both cases, allowing for inference of both region and function simultaneously. Modified generative architectures such as auto-regressive generative models (Shin *et al.* 2021), or masked diffusion models (Hallee *et al.* 2025) would allow for the calculation of entropy jointly over entire regions, potentially addressing current difficulties in modeling difficult targets such as the LLPS dataset.

## 5 Conclusion

In this work, we formalize semi-supervised functional residue annotation through a benchmark suite of eight datasets. We demonstrate that while entropy from pre-trained language models is a strong baseline for conserved residue identification, it may be outperformed by a function-conditioned generative model. When trained with a novel context-span masking objective, this model excels at retrieving broader functional regions like domains and repeats. We also introduce entropy gain, calculated as $g_{\theta ,\theta_{\mathrm{baseline}}}(R_{i})=h_{\theta_{\mathrm{baseline}}}(R_{i})-h_{\theta }(R_{i})$, as a method to specifically isolate the impact of functional information on a residue’s predicted distribution. This gain metric provides a distinct signal of functional relevance, independent of sequence conservation.

The overall pipeline offers a purely semi-supervised framework for identifying functionally important domains, motifs, or sites within proteins of interest. In the future we hope to see successes in function-conditioned modeling applied directly to the guidance of residue-level protein experiments, or even direct protein engineering through residue optimization.

## Supplementary Material

btag642_Supplementary_Data

## Data Availability

Source code for constructing the benchmark datasets, fine-tuning the GO classifier and function-conditioned ESM-C models, and computing all entropy, entropy-gain, and interpretability scores is available at github.com/mofradlab/go_interp, with an archived snapshot of the code and the eight residue-level benchmark datasets (CSA, InterPro active site, InterPro binding site, ELMS, BioLiP, LLPS, InterPro repeat, and InterPro domain) deposited at Zenodo (doi.org/10.5281/zenodo.21401001). All source data are publicly available: protein sequences and Gene Ontology annotations were obtained from SwissProt and the Gene Ontology Annotation database via gobench.org (Dickson *et al.* 2023); residue-level annotations were obtained from the Mechanism and Catalytic Site Atlas (Ribeiro *et al.* 2018), InterPro and interpro2go (Hunter *et al.* 2009), BioLiP2 (Zhang *et al.* 2024), the Eukaryotic Linear Motif resource (Kumar *et al.* 2019), and PhaSePro (Mészáros *et al.* 2020); homologous sequences for PSSM construction were drawn from UniRef90 (Suzek *et al.* 2015). The pre-trained ESM-Cambrian model is available from EvolutionaryScale (Hayes *et al.* 2024).
