# Supplementary material for: Semi-supervised retrieval of functional residues through the integration of protein language models and gene ontology data
Source: Bioinformatics. 2026 Aug 31;42(9):btag642. doi: 10.1093/bioinformatics/btag642 (PMC13631926; doi:10.1093/bioinformatics/btag642)
Supplement: Supplementary file 1 [file btag642_Supplementary_Data.pdf]

S1 Dataset Construction for Semi-supervised Residue Extraction

Each dataset provides a set of proteins with annotated functional residues or regions, paired with Gene Ontology (GO) term labels. Sequences longer than 850 amino acids are excluded from all datasets to limit computational cost.

S1.1 Catalytic Site Atlas (CSA)

Mechanistic annotations are drawn from the M-CSA curated data file (`curated_data.csv`), which lists the residue indices of catalytic residues for each enzyme along with its EC number. EC numbers are mapped to GO terms via the `ec2go` file from the Gene Ontology resource. For each UniProt accession, the set of annotated catalytic residue indices is aggregated across all M-CSA entries sharing that accession, and the corresponding protein sequence is fetched from UniProt. Proteins for which no EC-to-GO mapping exists are discarded. The resulting dataset records, for each protein, its catalytic residue indices and associated GO terms.

S1.2 BioLiP

The BioLiP non-redundant ligand–protein interaction dataset (`BioLiP_nr.txt`) is downloaded together with the corresponding non-redundant protein FASTA file. Entries are restricted to the nine most frequently occurring ligand types: ADP, CA, CLA, MG, MN, ZN, DNA, peptide, and RNA. Up to 120 proteins are sampled per ligand type. Binding-site residue indices are extracted from the renumbered (sequential) residue field and converted to integer indices. Only entries that carry at least one GO term annotation are retained. Each dataset entry records a protein's binding-site residue indices, its GO terms, and the cognate ligand type.

S1.3 Eukaryotic Linear Motifs (ELMs)

Instance and class files are downloaded from the ELM database together with a per-ELM GO-term mapping. Each ELM instance provides the UniProt accession of the host protein and the start/end positions of the linear motif. These positions are stored as `(start, end)` index tuples. ELM entries are merged with the GO-term table so that each instance carries the GO terms associated with its ELM class. Entries sharing the same UniProt accession and GO-term set are grouped, and their annotated intervals are collected into a list. To control dataset size, only one representative protein is retained per ELM class identifier. Protein sequences are fetched from UniProt in batch. Each entry records a protein, its list of annotated motif intervals, and the associated GO terms.

S1.4 Liquid–Liquid Phase Separation (LLPS)

Phase-separating proteins and their LLPS-driving regions are obtained from the PhasePro database (`phasepro.json`). Each entry specifies the boundaries of the region sufficient for phase separation, given as semicolon-delimited `start--end` ranges, as well as the subcellular organelle(s) in which phase separation occurs. Organelle names are mapped to GO cellular-component terms by exact name and synonym matching against the GO basic ontology (via `goatools`); a hand-curated fallback dictionary covers organelle descriptions that do not match any GO term name directly. Annotated boundaries are parsed into `(start, end)` tuples. Each entry records a protein, its phase-separating region boundaries, and the derived GO terms.

S1.5 InterPro Domains

The InterPro XML release is parsed to obtain all entries with more than 10,000 annotated proteins. GO-term annotations are loaded from the `interpro2go` mapping. Entries are partitioned by InterPro type (Domain, Repeat, Active site, Binding site, Homologous superfamily, Family), and up to 12 representative InterPro accessions are sampled per type. For each selected accession, matching proteins are identified from the `protein2ipr` mapping, restricted to Swiss-Prot sequences. Entries in which the annotated region spans more than 60% of the protein length are removed so that the labeled region is genuinely a subsequence of the protein. Up to 40 proteins are sampled per InterPro accession. Each entry records a UniProt accession, the `(start, end)` coordinates of the InterPro domain, the GO terms for that accession, the InterPro type, and the full protein sequence.

S2 Training Methodology

The following outlines the computational methodology used to train the models described in the main paper. Our work centered on two main modeling approaches: (1) a Gene Ontology (GO) classifier, which was fine-tuned to serve as a basis for evaluating classifier interpretability methods, and (2) a function-conditioned generative model, developed to directly integrate functional information into residue-level predictions. All models were implemented using the PyTorch and PyTorch Lightning frameworks.

S2.1 Gene Ontology (GO) Classifier for Interpretability

To establish a baseline and evaluate the efficacy of feature attribution techniques, we fine-tuned a protein language model for multi-label GO term classification.

The classifier was built upon the pre-trained 600M ESM-Cambrian (ESM-C) protein language model. For a given input sequence, the final-layer residue

embeddings from the ESM-C encoder were subjected to mean pooling to produce a single, fixed-size vector representing the entire protein. This sequence-level representation was then fed into a linear classification head to produce logits for each GO term in our dataset.

S2.2 Training and Optimization

We adopted a two-phase fine-tuning strategy on our curated SwissProt-GOA dataset. 1. Phase 1 (Head Training): The parameters of the ESM-C base model were frozen, and only the final linear classification head was trained. This phase used a higher learning rate to quickly adapt the new layer to the task. 2. Phase 2 (Full Model Fine-tuning): The entire model, including the ESM-C encoder, was unfrozen and trained for five epochs. We used the AdamW optimizer with a learning rate that was annealed from 1×10^{-5} down to 1×10^{-6}.

The model was trained by minimizing the Binary Cross-Entropy (BCE) loss, and its performance was monitored using the micro F1-score on a held-out validation set.

S2.3 Function-Conditioned Generative Model

This model was developed to guide the generative process of a protein language model with functional annotations, with the goal of improving the identification of functionally relevant residues.

S2.3.1 Model Architecture

The architecture augments the pre-trained ESM-C model. A learnable GO term embedding matrix, $E_{GO} \in \mathbb{R}^{|\mathcal{G}| \times d_{emb}}$, was introduced, where $|\mathcal{G}|$ is the total number of GO terms and d_{emb} is the model's embedding dimension. For a protein annotated with a set of GO terms represented by a multi-hot vector y, an aggregate function vector v_{func} was computed by summing the corresponding embedding vectors:

$$v_{func} = \sum_{j=1}^{|\mathcal{G}|} y_j E_{GO}^{(j)}$$

This vector was then passed through a linear projection and added to each residue embedding at the input layer of the ESM-C transformer. This allows the model to condition its sequence predictions on the global functional context provided.

S3 MSA Baseline Pipeline

The MSA baseline uses position-specific conservation from multiple sequence alignments (MSAs) as a position-level score, compared against model-predicted entropy. For each query protein, we search a large sequence database (UniRef)

for homologs, assemble an MSA, and compute per-position Shannon entropy from the resulting position-specific scoring matrix (PSSM). Lower entropy indicates higher conservation, which serves as a proxy for functional importance.

MSA baselines are computed for four evaluation datasets: **CSA**, **LLPS**, **ELMs**, and **IP Domain**.

S3.1 Step 1: Extract Query FASTAs

Script: `build_fasta.ipynb`

Each evaluation dataset CSV is read from `gen_datasets/datasets/` and converted to a FASTA file. Sequence IDs follow two conventions:

- **CSA, LLPS, ELMs:** UniProt accession (e.g. `P12345`)
- **IP Domain:** Integer index (`ipdom0`, `ipdom1`, ...), used to avoid colon characters in some UniProt IDs that cause downstream issues

Output: `gen_datasets/datasets/{dataset}.fasta`

S3.2 Step 2: Homolog Search with MMseqs2

Script: `run_mmseqs2.sh`

Each query FASTA is searched against a GPU-accelerated UniRef database using MMseqs2 with two search iterations. The output is an alignment file in M8 (BLAST tabular) format containing columns: `query, target, fident, pident, nident, qlen, tlen, qstart, qend, tstart, tend, evalue, bits, qseq, tseq`.

```
# Run from go_ml/msa_pipeline/
bash run_mmseqs2.sh [GPU_ID]
```

MMseqs2 commands per dataset:

```
mmseqs createdb {dataset}.fasta query_db/{dataset}_db
mmseqs search query_db/{dataset}_db uniref_data/
    uniref_db_gpu \
     results_db/{dataset}_result_db tmp --gpu 1 --num-
         iterations 2
mmseqs convertalis query_db/{dataset}_db uniref_data/
    uniref_db_gpu \
     results_db/{dataset}_result_db {dataset}_aln.m8 \
     --format-output "query,target,...,qseq,tseq"
```

Output: `{dataset}_aln.m8` (one file per dataset)

S3.3 Step 3: Parse Hits into Per-Protein FASTAs

Script: `build_homologues.ipynb`

The M8 alignment files are parsed and filtered to produce one unaligned FASTA file per query protein containing its homologous sequences. Filtering criteria:

- Percent identity $\leq 95\%$ (exclude near-identical sequences)
- Homolog length within 2σ of the mean length across all hits
- Maximum homolog length ≤ 1500 AA
- Maximum 450 homologs retained per query (truncated by rank)
- Query sequence is appended as the seed if not already present
- Queries with fewer than 5 homologs after filtering are skipped

Output: `uniref_msa/{dataset}_msa/{protein_id}_homologues.fasta`

S3.4 Step 4: Multiple Sequence Alignment with MUSCLE

Each per-protein FASTA (unaligned homologs) is aligned using MUSCLE v5:

```
# Single protein
muscle -align uniref_msa/csa_msa/P12345_homologues.fasta \
       -output uniref_msa/csa_msa_output/
           P12345_homologues_aligned.fasta

# Batch (all proteins in a dataset)
for f in uniref_msa/csa_msa/*.fasta; do
    name=$(basename "$f" .fasta)
    muscle -align "$f" \
           -output "uniref_msa/csa_msa_output/${name}
               _aligned.fasta"
done
```

Output: `uniref_msa/{dataset}_msa_output/{protein_id}_homologues_aligned.fasta`

S3.5 Step 5: Compute PSSM Entropy

Script: `dataset_eval/msa_gen.ipynb`

Aligned FASTAs are loaded and converted to position-specific scoring matrices (PSSMs). Per-position Shannon entropy is computed over the 20 amino acid frequency distributions:

$$H_i = -\sum_{a=1}^{20} p_{i,a} \log p_{i,a}$$

where $p_{i,a}$ is the frequency of amino acid a at position i across the MSA. Columns where $\geq 30\%$ of sequences have a gap are assigned maximum entropy and excluded from evaluation. The resulting entropy vector is saved alongside the protein ID list and sequence length mask.

S3.5.1 Training Objectives and Masking Strategies

The model's parameters (the ESM-C base and the new function embeddings) were jointly fine-tuned using a masked language modeling (MLM) objective. We experimented with two distinct masking schemes:

In the default training, following the original BERT methodology, 15% of the residues in each sequence were randomly selected and replaced with a '[MASK]' token. In Context-Span Masking, the objective is made more challenging to increase the relevance of functional information. For each sequence, a random, contiguous 100-residue "focus region" was defined. All residues outside this region were masked and excluded from the loss calculation. Within the focus region, contiguous spans of 2 to 5 residues were masked until 30% of the total sequence residues were masked. The model was trained to predict the original amino acids for these masked spans.

S3.5.2 Training and Optimization

The function-conditioned model was fine-tuned for five epochs using the AdamW optimizer. The learning rate was annealed from an initial value of 1×10^{-5} to a final value of 1×10^{-6}. The training loss was the standard cross-entropy loss calculated over the masked positions. A baseline model (standard ESM-C without functional conditioning) was also fine-tuned under identical conditions for comparison.

S4 Derivation of Residue Importance Scores

To evaluate the models, we derived a per-residue importance score from each.

S4.1 Positional Entropy

For a given sequence, a Position-Specific Scoring Matrix (PSSM) was estimated by repeatedly masking each position and averaging the model's output probability distributions for that position. The final importance score was the Shannon entropy of this predicted distribution, where lower entropy signifies higher conservation and thus greater importance.

From Function-Conditioned Models (Entropy Gain): To isolate the contribution of the functional information, we calculated the entropy gain. This metric is defined as the reduction in positional entropy when functional conditioning is applied: $g_{\theta,\theta_{baseline}}(R_i) = h_{\theta_{baseline}}(R_i) - h_\theta(R_i)$. A positive gain indicates that the functional context made the model more certain about a residue's identity.

S4.2 Masking Strategies

Estimating PSSMs from our generative models requires us to repeatedly mask and demask protein residues to predict their conservation. We evaluate strate-

gies of both randomly masking a percentage of residues for each demasking iteration and iterating through individual residues and masking only them. We also evaluate a span-masking strategy in which as sliding window of masked residues is run across the protein.

For percentage masking trials, residues may be evaluated any number of times. We test coverage levels of 1, 6, and 12, to evaluate the effect of repeated trials.

Table 1: Performance metrics for the CSA dataset. MRR, AUC, BAUC, and Top-30 metrics reported. BAUC represents AUC values of ROC curve over all residues.

Method	Perc	Coverage	MRR	AUC	BAUC	Top-30 Score
Percentage Masking	0.05	1	0.1336	0.7980	0.7804	0.5484
Percentage Masking	0.05	6	0.4100	0.8147	0.7954	0.5940
Percentage Masking	0.05	12	0.4091	0.8153	0.7956	0.5961
Percentage Masking	0.15	1	0.0401	0.7308	0.7173	0.1160
Percentage Masking	0.15	6	0.4154	0.8160	0.7954	0.5946
Percentage Masking	0.15	12	0.4130	0.8158	0.7952	0.5970
Percentage Masking	0.3	1	0.0415	0.7315	0.7162	0.1289
Percentage Masking	0.3	6	0.4112	0.8145	0.7917	0.5913
Percentage Masking	0.3	12	0.4142	0.8167	0.7932	0.5966
Indiv Masking	-	-	0.4088	0.8147	0.7953	0.5950
Neighborhood 5 Masking	-	-	0.4040	0.7999	0.7749	0.5467
Naive	-	-	0.0589	0.5006	0.5005	0.0909

Table 2: Performance metrics for the IP domain dataset. MRR, AUC, BAUC, and Top-30 metrics reported. BAUC represents AUC values of ROC curve over all residues.

Method	Perc	Coverage	MRR	AUC	BAUC	Top-30 Score
Percentage Masking	0.05	1	0.2651	0.7575	0.7525	0.5256
Percentage Masking	0.05	6	0.7827	0.7801	0.7730	0.6879
Percentage Masking	0.05	12	0.7826	0.7801	0.7732	0.6899
Percentage Masking	0.15	1	0.1552	0.6665	0.6742	0.1602
Percentage Masking	0.15	12	0.7834	0.7865	0.7789	0.7008
Percentage Masking	0.15	6	0.7848	0.7858	0.7783	0.6974
Percentage Masking	0.3	1	0.1572	0.6759	0.6816	0.1631
Percentage Masking	0.3	6	0.7896	0.7933	0.7856	0.7101
Percentage Masking	0.3	12	0.7875	0.7944	0.7865	0.7084
Indiv Masking	-	-	0.7753	0.7771	0.7699	0.6837
Neighborhood 5 Masking	-	-	0.8185	0.8099	0.7990	0.7417
Naive	-	-	0.5026	0.4959	0.5004	0.3237

S4.3 Context-Span Masking

Table 3: Results for varying context length and span length parameters for function-conditioned fine-tuning.

Context Length					Span-Masking Length				
Cont. Len	Span Len	MRR	AUC	Top-30	Cont. Len	Span Len	MRR	AUC	Top-30
**CSA**									
50	5	0.250	0.692	0.321	100	2	0.319	0.750	0.435
100	5	0.258	0.730	0.377	100	5	0.258	0.730	0.377
200	5	0.260	0.738	0.387	100	10	0.233	0.704	0.305
**LLPS**									
50	5	0.334	0.512	0.319	100	2	0.302	0.478	0.294
100	5	0.305	0.470	0.293	100	5	0.305	0.470	0.293
200	5	0.328	0.461	0.292	100	10	0.330	0.489	0.299
**ELMS**									
50	5	0.049	0.414	0.053	100	2	0.053	0.397	0.053
100	5	0.045	0.385	0.055	100	5	0.045	0.385	0.055
200	5	0.051	0.377	0.055	100	10	0.043	0.380	0.046
**BioLiP**									
50	5	0.266	0.635	0.323	100	2	0.283	0.669	0.371
100	5	0.246	0.661	0.342	100	5	0.246	0.661	0.342
200	5	0.233	0.663	0.328	100	10	0.221	0.644	0.317
**IP Repeat**									
50	5	0.453	0.681	0.286	100	2	0.458	0.670	0.269
100	5	0.357	0.664	0.261	100	5	0.357	0.664	0.261
200	5	0.308	0.649	0.226	100	10	0.376	0.658	0.259
**IP Domain**									
50	5	0.790	0.836	0.755	100	2	0.824	0.832	0.725
100	5	0.687	0.829	0.710	100	5	0.687	0.829	0.710
200	5	0.723	0.857	0.734	100	10	0.717	0.849	0.729
**IP Binding Site**									
50	5	0.417	0.722	0.342	100	2	0.429	0.709	0.348
100	5	0.356	0.714	0.322	100	5	0.356	0.714	0.322
200	5	0.336	0.725	0.271	100	10	0.316	0.718	0.324
**IP Active Site**									
50	5	0.430	0.833	0.450	100	2	0.501	0.857	0.484
100	5	0.474	0.842	0.452	100	5	0.474	0.842	0.452
200	5	0.388	0.795	0.350	100	10	0.324	0.816	0.395

S4.4 Semi-supervised Training Epochs

For functional conditioning training, we evaluate the effect of training epoch on the quality of residue importance scores. For the CSA and IP-Domain datasets, we report results for epochs 1, 2, 5, and 7, as well as the best-performing epoch

as recorded by validation loss for each dataset and metric.

We find overall that performance is maximized for both the CSA and IP-Domain datasets at early epochs of training, with a general decline in performance as training progresses to late epochs. This suggests that the model may be overfitting to the relatively small quantity of functionally annotated training data and losing general knowledge from pre-training. We employ cosine annealing of the learning rate, which diminishes the magnitude of parameter updates as training progresses, limiting the overall decline from extended training.

In practice validation masking loss plateaus quickly, in the second or third epochs of training. We find that downstream functional residue prediction performance plateaus and begins a very gradual decline around the second epoch of training, indicating that early stopping is a good heuristic for selecting strong overall models. We apply early stopping for evaluation models in practice.

Table 4: Ablation results on the **CSA** dataset.

**Variant**	**Epoch**	**AUC**	**MRR**	**Top-30**
Span / Unfrozen	1	**0.7342**	0.3032	0.3824
	2	0.7330	**0.3058**	**0.3912**
	5	0.7330	0.2907	0.3888
	7	0.7285	0.2805	0.3751
	Best	0.7295	0.2763	0.3802
Span / Frozen	1	0.7299	0.2895	0.3683
	2	0.7314	0.2833	0.3846
	5	0.7295	0.2849	0.3847
	7	0.7270	0.2678	0.3745
	Best	0.7303	0.2677	0.3803
Perc / Unfrozen	1	0.7170	0.2697	0.3479
	2	0.7179	0.2661	0.3481
	5	0.7122	0.2580	0.3312
	7	0.7066	0.2462	0.3224
	Best	0.7113	0.2441	0.3299
Perc / Frozen	1	0.7199	0.2733	0.3488
	2	0.7166	0.2565	0.3444
	5	0.7135	0.2425	0.3372
	7	0.7106	0.2443	0.3284
	Best	0.7155	0.2530	0.3367

S4.5 Residue Attention Weights

In our evaluation, we estimate residue importance through the sum of ingoing attention from all-head in a given layer of the ESM-C encoder. We apply this process to five layers from the ESM-C model, finding that the strongest overall

Table 5: Ablation results on the **IP-Domain** dataset.

**Variant**	**Epoch**	**AUC**	**MRR**	**Top-30**
Span / Unfrozen	1	0.8328	**0.7386**	**0.7174**
	2	0.8307	0.7232	0.7112
	5	0.8323	0.7270	0.7130
	7	0.8327	0.7176	0.7173
	Best	0.8310	0.7301	0.7146
Span / Frozen	1	**0.8377**	0.7214	0.7170
	2	0.8308	0.7133	0.7034
	5	0.8303	0.7177	0.7125
	7	0.8317	0.7166	0.7090
	Best	0.8357	0.7211	0.7140
Perc / Unfrozen	1	0.8182	0.7222	0.7089
	2	0.8207	0.7110	0.7087
	5	0.8247	0.7075	0.7107
	7	0.8266	0.7050	0.7123
	Best	0.8265	0.7097	0.7062
Perc / Frozen	1	0.8205	0.7362	0.7136
	2	0.8237	0.7185	0.7100
	5	0.8229	0.7180	0.7085
	7	0.8270	0.7037	0.7095
	Best	0.8244	0.7166	0.7096

results are delivered by attention patterns within a late-intermediate layer for almost all datasets and metrics. Below, we report the complete set of results.

S4.6 Attention-Layer Evaluation

Table 6: Performance of individual attention layers for residue-level prediction across datasets.

Layer	MRR	AUC	Top-30
**CSA**			
0	0.080	0.621	0.138
9	0.055	0.495	0.065
18	0.076	0.640	0.162
27	0.080	0.587	0.131
35	0.087	0.359	0.103
**LLPS**			
0	0.452	0.506	0.428
9	0.350	0.383	0.285

Continued on next page

Table 6 (continued)

Layer	MRR	AUC	Top-30
18	0.386	0.482	0.380
27	0.426	0.451	0.276
35	0.468	0.484	0.326
**ELMS**			
0	0.105	0.444	0.101
9	0.078	0.456	0.076
18	0.114	0.518	0.126
27	0.114	0.488	0.093
35	0.102	0.513	0.093
**BioLiP**			
0	0.133	0.597	0.199
9	0.086	0.472	0.122
18	0.121	0.611	0.230
27	0.120	0.570	0.200
35	0.138	0.395	0.132
**IP Repeat**			
0	0.212	0.475	0.114
9	0.362	0.525	0.173
18	0.424	0.542	0.195
27	0.408	0.588	0.188
35	0.343	0.558	0.182
**IP Homologous Superfamily**			
0	0.586	0.503	0.250
9	0.338	0.508	0.349
18	0.549	0.496	0.314
27	0.538	0.520	0.305
35	0.412	0.499	0.267
**IP Domain**			
0	0.308	0.506	0.278
9	0.782	0.628	0.425
18	0.847	0.580	0.306
27	0.799	0.624	0.460
35	0.509	0.507	0.457
**IP Binding Site**			
0	0.237	0.534	0.162
9	0.373	0.486	0.133
18	0.343	0.585	0.168
27	0.305	0.617	0.161
35	0.243	0.434	0.144
**IP Active Site**			
0	0.146	0.537	0.110
9	0.260	0.526	0.111

Continued on next page

Table 6 (continued)

Layer	MRR	AUC	Top-30
18	0.348	0.664	0.206
27	0.386	0.672	0.200
35	0.181	0.343	0.095

S4.7 Interpretability

From the GO Classifier (Interpretability Methods): We applied four feature attribution methods to the fine-tuned GO classifier: Layer-wise Integrated Gradients (LIG), Layer-wise Gradient X Activation (LGA), Layer-wise DeepLIFT (LDA), and Layer-wise GradientShap (LGS). For each method, the L2 norm of the attribution values for a residue's input embedding was taken as its final importance score.

S5 Multi-GO-Term Attribution Aggregation

In the main text, each interpretability method attributes residue importance with respect to the single most-specific (deepest) GO term associated with an annotated region. Because a region may be annotated with several GO terms, we tested whether aggregating attributions across the full set of positive GO terms—the annotated term(s) together with all of their GO ancestors—recovers functional residues more effectively. For each protein we computed per-residue attributions for every positive GO term and combined them by either averaging (*Mean*) or taking the element-wise maximum (*Max*) across terms, then re-evaluated retrieval against the most-specific-term baseline on all nine residue-level benchmarks.

Averaged over the benchmarks (Table 7), mean aggregation yields a small, consistent improvement in AUC for every method, but the effect is marginal: the largest averaged gain is +0.014 AUC (Gradient×Activation), and the improvement does not carry over reliably to the ranking-based metrics (MRR, Top-30). Max aggregation is essentially neutral ($|\Delta\text{AUC}| \leq 0.004$ for every method). The per-dataset breakdowns (Tables 8–16) confirm that the direction of the effect is inconsistent: aggregation helps modestly on some datasets (e.g. CSA, ELMs) but is flat or slightly negative on others (e.g. InterPro Repeat, Homologous Superfamily). Crucially, aggregation does not change the relative ordering of interpretability methods or any conclusion drawn in the main text. We therefore retain the most-specific-term attribution throughout, as it is the simplest choice and the most informative single target for the classifier, while noting that averaging over positive terms offers a slight edge in AUC where a small gain is worthwhile.

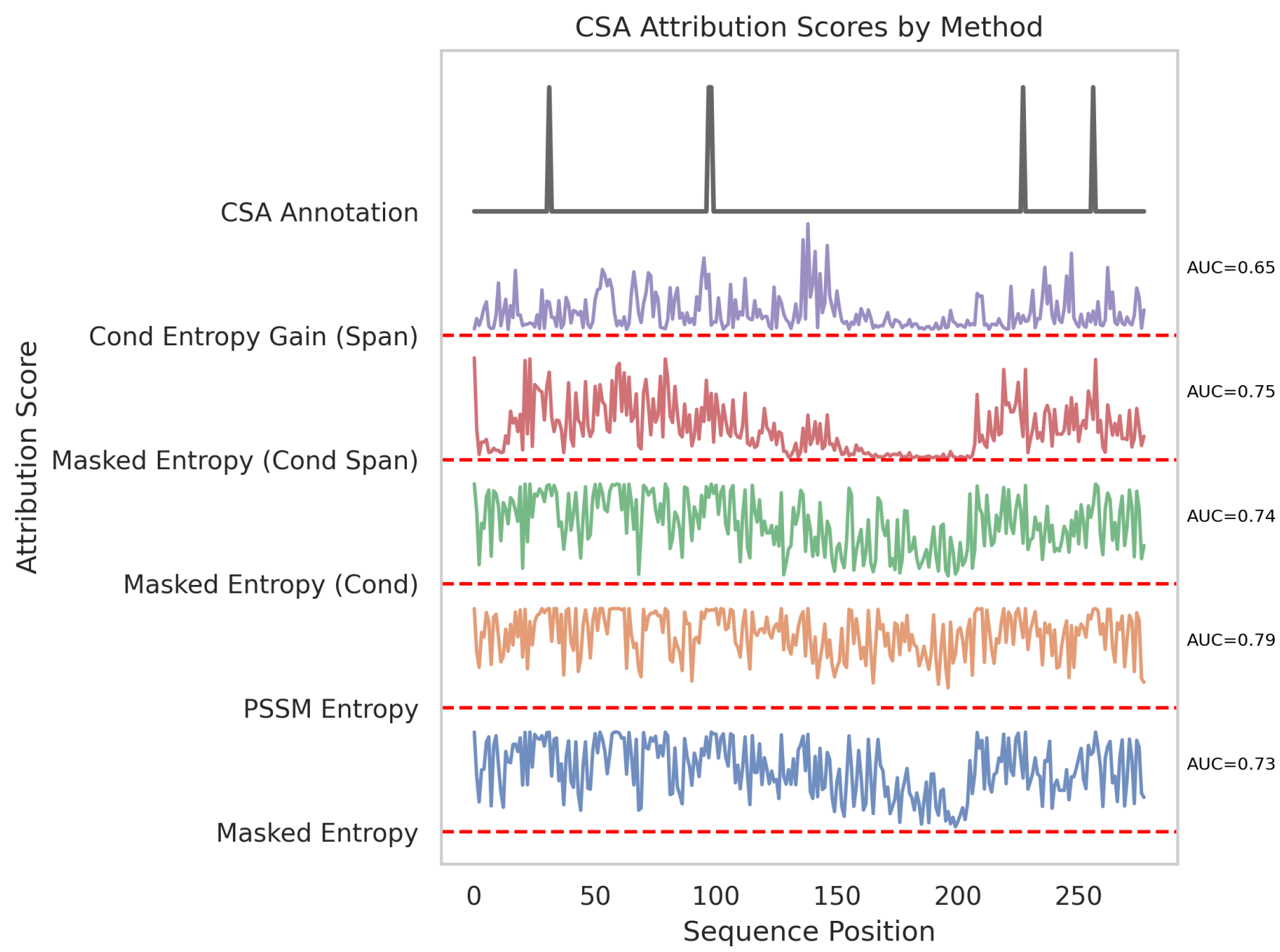

Figure 1: Case study of residue entropy based methods predicting residue importances of Uniprot protein O31168. Residue number plotted against normalized importance scores corresponding to negative entropy in the case of entropy based methods, or reduction in entropy in the case of entropy gain methods.

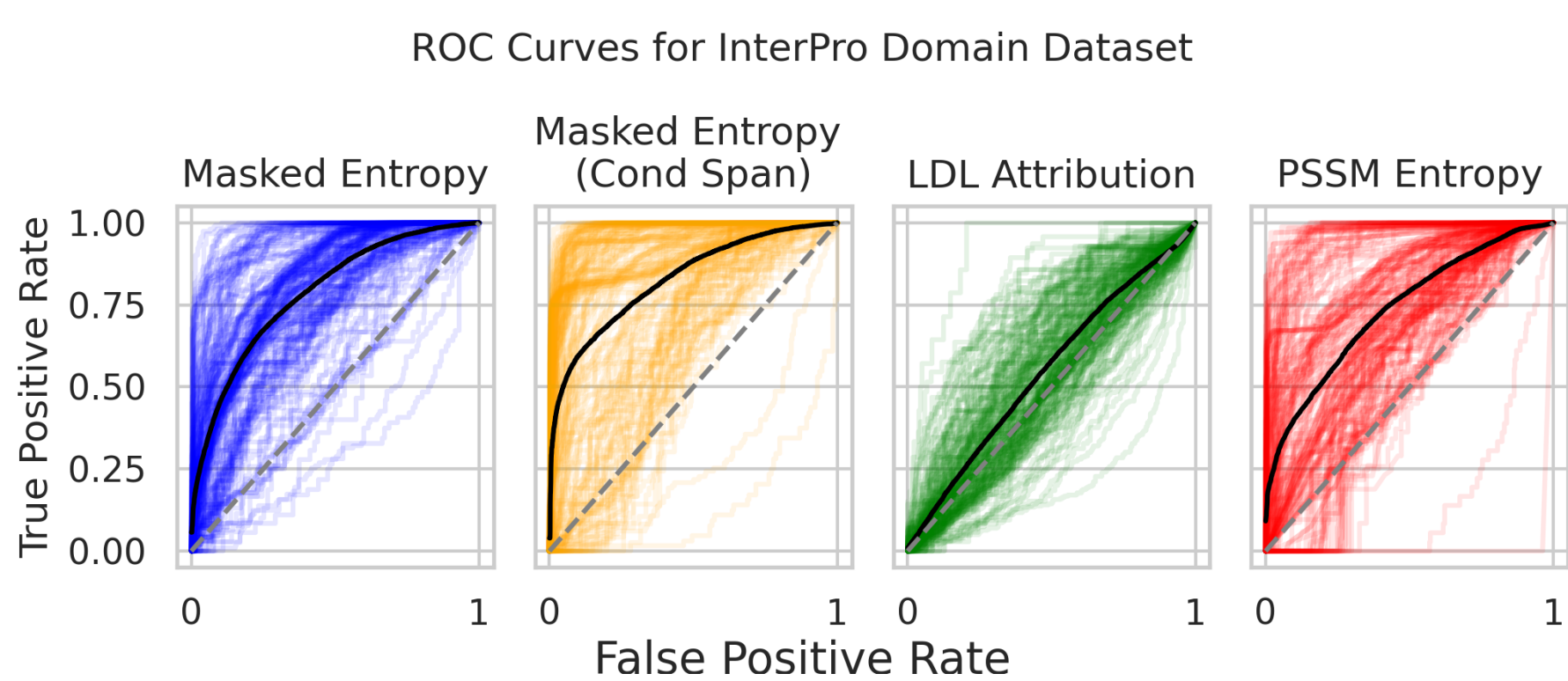

Figure 2: ROC curves for domain retrieval from the InterPro domain dataset on each dataset protein, plotted for the masked entropy, masked entropy with context-span masking and functional conditioning, LDL attribution, and PSSM entropy.

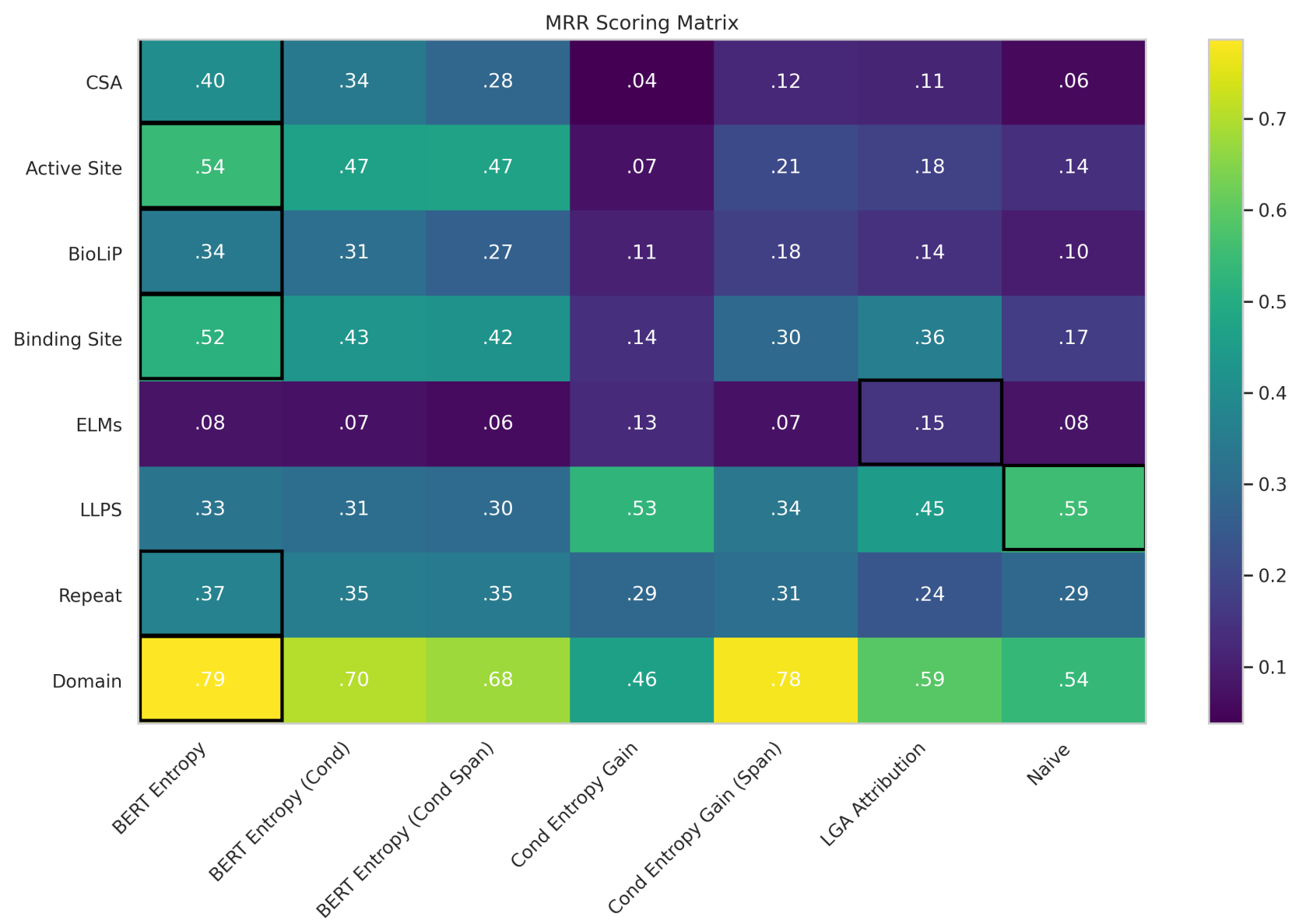

Figure 3: A) All-dataset comparison of Masked Entropy, Function-Conditioned Entropy, and interpretability methods on retrieval of functionally important residues, as evaluated by Mean Reciprocal Rank.

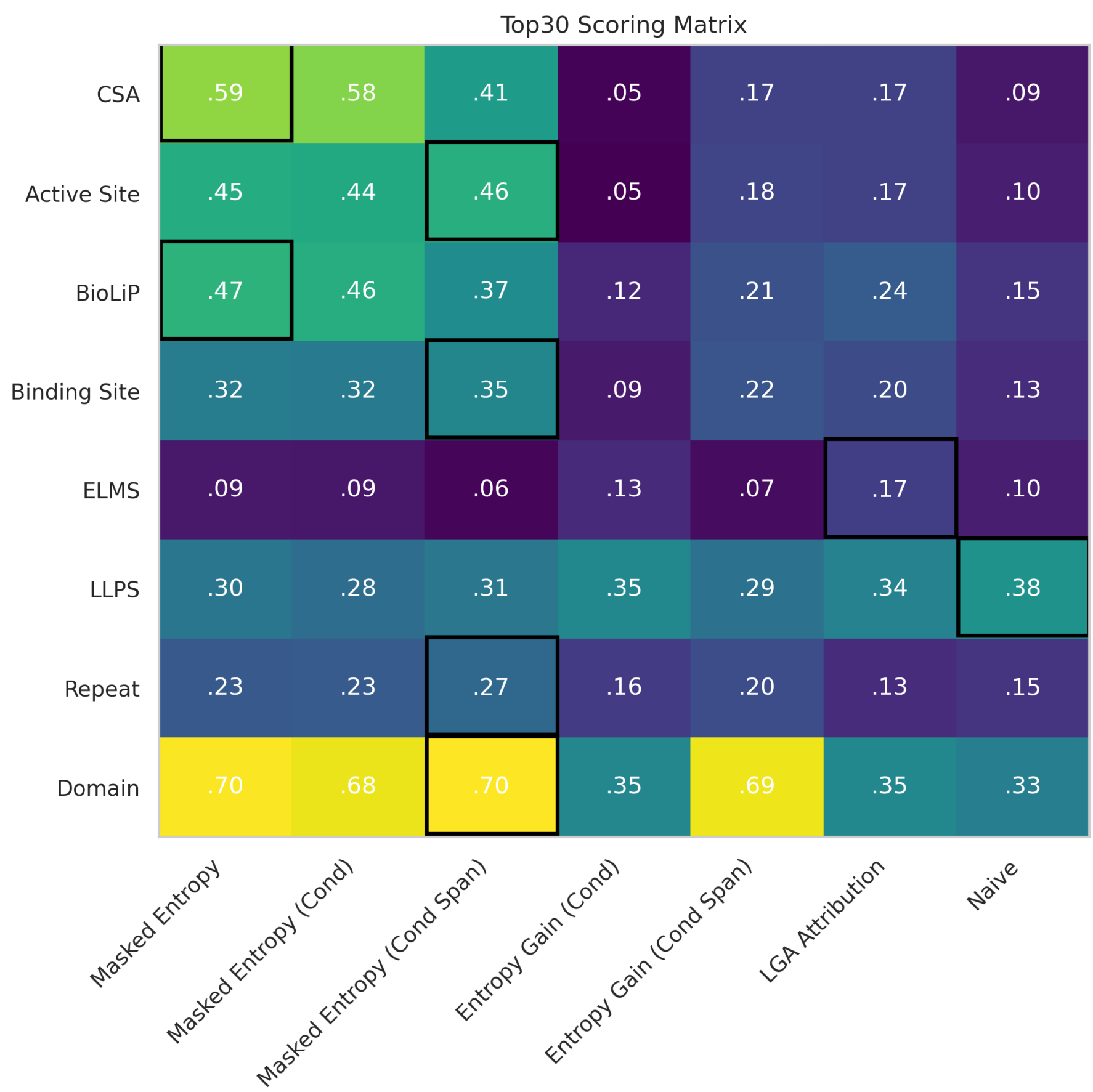

Figure 4: A) All-dataset comparison of Masked Entropy, Function-Conditioned Entropy, and interpretability methods on retrieval of functionally important residues, as evaluated by mean of Top30 metric across dataset.

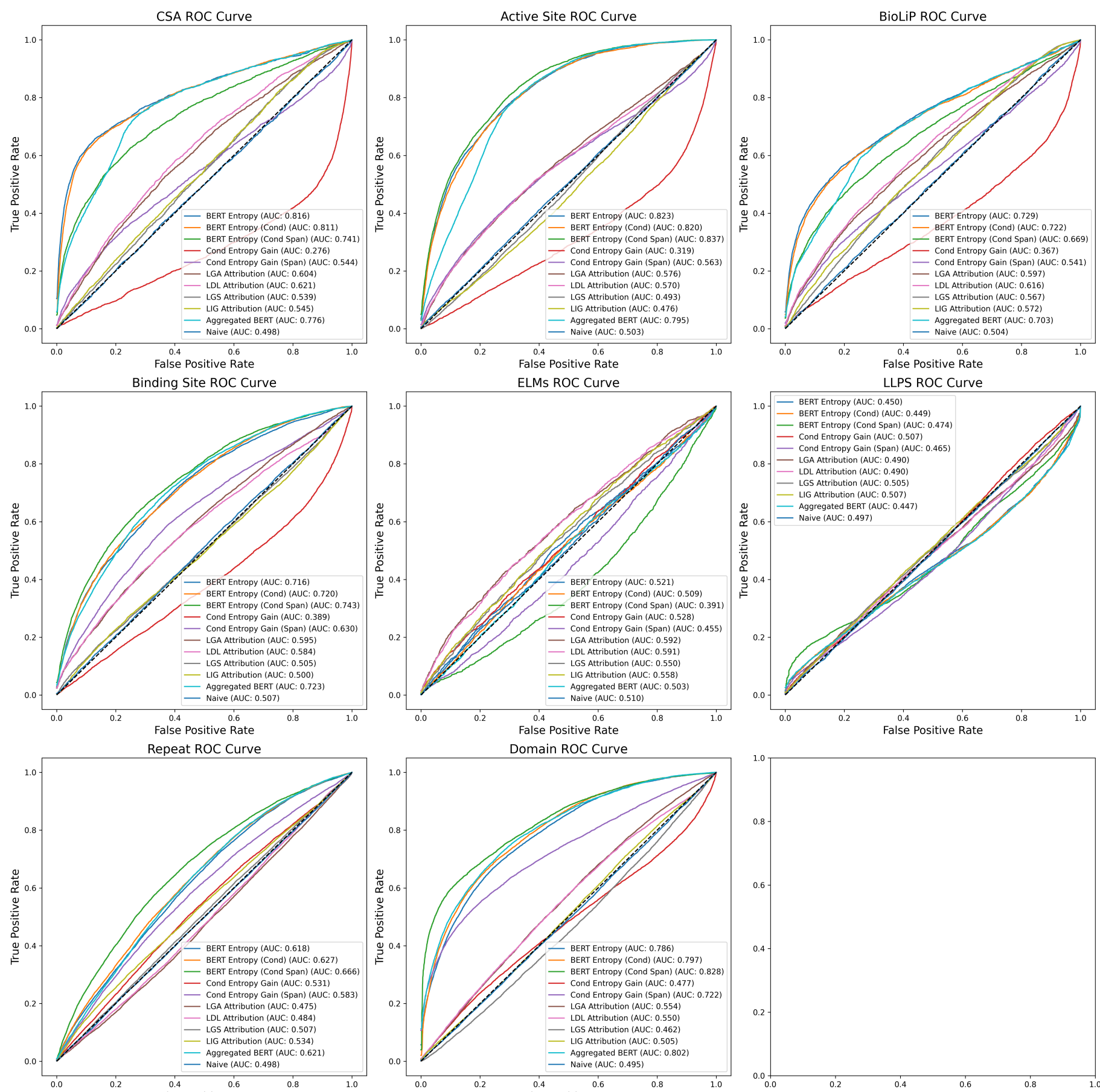

Figure 5: Plots of meanROC curves for all models on all datasets.

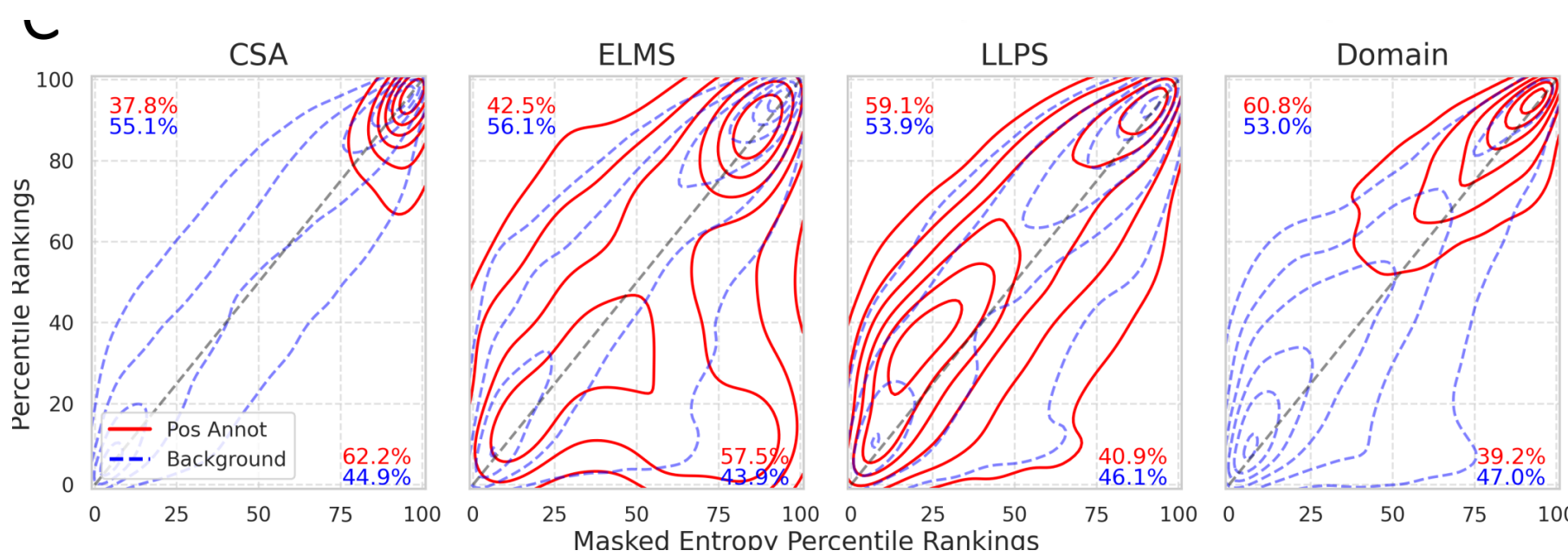

Figure 6: Topographic maps of percentile scoring by ESM-C masked entropy vs. ESM-C fine-tuned with a functional context on a context-span masking loss. For each sequence in our dataset, percentile ranking of each residue as scored by Masked Entropy and Masked Entropy with functional conditioning and context-span masking are calculate. We plot the density of residues from all sequences, inversely weighted by sequence length, on the space of pairs of percentile importance assignments from the two models. Density plots for annotated and baseline residues plotted separately, with percentage of residues for which entropy rank is higher than span-context entropy rank labeled in lower-right, and inverse labeled in upper-left.

S6 Stratification by GO-Term Depth

One reviewer asked whether retrieval performance is confounded by the depth (specificity) of the target GO term, on the grounds that deeper terms are harder in the protein-level GO-*prediction* setting. In our task the GO term is a conditioning *input* rather than a prediction target, so this difficulty does not obviously transfer. To test it directly, we split each of the nine residue-level benchmarks at its median GO depth (the depth of the deepest annotated term, `GOTerm[0]`) into a low-depth and a high-depth subset, and recomputed all metrics within each subset. ΔAUC in Tables 17–25 is the high-depth AUC minus the low-depth AUC, so a negative value indicates that deeper (more specific) target terms are harder. In the row labels, attribution methods are annotated with the GO-term set they explain (*most-specific*, or *GO-mean*/*GO-sum*/*GO-max* aggregated over all positive terms), and the Cond-Span variants are labelled by their context-length/span-length setting (e.g. "100/5").

For our primary function-conditioned entropy models (Masked Entropy, Masked Entropy (Cond), and Masked Entropy (Cond Span)), there is *no systematic depth penalty*: averaged over the nine benchmarks, the high-versus-low ΔAUC lies within ± 0.013 of zero for all three variants, and its sign varies by dataset. On CSA (Table 17), the largest and one of the cleanest benchmarks (n=532/252 low/high), all three variants are flat to slightly *better* on deep terms (ΔAUC +0.007 to +0.010), with AUC near 0.81 in both strata; several other datasets (InterPro Binding Site, Homologous Superfamily, Repeat) show a clear high-

Table 7: Multi-GO-term attribution aggregation averaged over all nine residue-level benchmarks. Aggregating attributions over all positive GO terms (*Mean*) improves over the single most-specific-term baseline for every interpretability method.

**Method**	**Aggregation**	**AUC**	**MRR**	**Top-30**	**ΔAUC**
Gradient×Activation (LGA)	Most-specific (baseline)	0.5490	0.3008	0.2239	–
	Mean over GO terms	**0.5634**	0.3098	0.2285	+0.0144
	Max over GO terms	0.5531	0.2979	0.2211	+0.0041
DeepLift (LDL)	Most-specific (baseline)	0.5522	0.3006	0.2270	–
	Mean over GO terms	0.5632	**0.3199**	**0.2305**	+0.0111
	Max over GO terms	0.5537	0.3047	0.2209	+0.0015
Integrated Gradients (LIG)	Most-specific (baseline)	0.5203	0.2607	0.2054	–
	Mean over GO terms	0.5236	0.2631	0.2058	+0.0033
	Max over GO terms	0.5220	0.2585	0.2078	+0.0016
Conductance (LC)	Most-specific (baseline)	0.5222	0.2012	0.1869	–
	Mean over GO terms	0.5230	0.1873	0.1824	+0.0008
	Max over GO terms	0.5218	0.1969	0.1803	-0.0004
GradientSHAP (LGS)	Most-specific (baseline)	0.5086	0.1880	0.1720	–
	Mean over GO terms	0.5141	0.1711	0.1750	+0.0055
	Max over GO terms	0.5125	0.1870	0.1798	+0.0038

depth *gain*. The main exception is InterPro Domain (Table 22), where the entropy and masking models lose 0.07–0.14 AUC on deep terms (e.g. Masked Entropy (Cond Span): 0.874 → 0.766); notably, the gradient-attribution baselines move in the *opposite* direction on this dataset (+0.06 to +0.09), so the effect is method- and dataset-specific rather than a uniform "specificity is harder" trend. Two caveats temper the InterPro results: the high-depth subsets are small (Domain n=77, Active Site n=80), making per-stratum estimates noisier than on CSA or BioLiP, and for domain/family annotations GO depth is partly a proxy for domain type and size rather than intrinsic localization difficulty. Overall, the stratification supports our framing: depth-related difficulty from the GO-prediction setting does not translate into a consistent degradation for residue-level localization.

Table 8: Multi-GO-term attribution aggregation on the **CSA** dataset. *Most-specific* is the original baseline, which attributes only the single most specific GO term; *Mean*/*Max* aggregate attributions over all positive GO terms (annotated term(s) plus all GO ancestors). ΔAUC is relative to that method's own baseline.

**Method**	**Aggregation**	**AUC**	**MRR**	**Top-30**	**ΔAUC**
Gradient×Activation (LGA)	Most-specific (baseline)	0.6044	0.1143	0.1723	–
	Mean over GO terms	0.6388	0.1393	0.1926	+0.0343
	Max over GO terms	0.6154	0.1322	0.1723	+0.0109
DeepLift (LDL)	Most-specific (baseline)	0.6206	0.1177	0.1873	–
	Mean over GO terms	**0.6496**	**0.1437**	**0.2040**	+0.0290
	Max over GO terms	0.6263	0.1346	0.1873	+0.0056
Integrated Gradients (LIG)	Most-specific (baseline)	0.5449	0.0628	0.1114	–
	Mean over GO terms	0.5504	0.0640	0.1253	+0.0055
	Max over GO terms	0.5460	0.0659	0.1235	+0.0011
Conductance (LC)	Most-specific (baseline)	0.6035	0.0667	0.1210	–
	Mean over GO terms	0.6021	0.0678	0.1257	-0.0015
	Max over GO terms	0.6045	0.0756	0.1273	+0.0009
GradientSHAP (LGS)	Most-specific (baseline)	0.5448	0.0597	0.0950	–
	Mean over GO terms	0.5424	0.0653	0.1144	-0.0024
	Max over GO terms	0.5323	0.0629	0.1056	-0.0126

Table 9: Multi-GO-term attribution aggregation on the **LLPS** dataset. *Most-specific* is the original baseline, which attributes only the single most specific GO term; *Mean*/*Max* aggregate attributions over all positive GO terms (annotated term(s) plus all GO ancestors). ΔAUC is relative to that method's own baseline.

**Method**	**Aggregation**	**AUC**	**MRR**	**Top-30**	**ΔAUC**
Gradient×Activation (LGA)	Most-specific (baseline)	0.4898	0.4520	0.3377	–
	Mean over GO terms	0.4868	0.4620	0.3224	-0.0030
	Max over GO terms	0.4910	0.4498	0.3235	+0.0012
DeepLift (LDL)	Most-specific (baseline)	0.4897	0.3801	0.3244	–
	Mean over GO terms	0.4882	0.4529	0.3131	-0.0015
	Max over GO terms	0.4916	0.4263	0.3084	+0.0019
Integrated Gradients (LIG)	Most-specific (baseline)	0.5069	**0.5008**	**0.3857**	–
	Mean over GO terms	0.5151	0.4788	0.3719	+0.0082
	Max over GO terms	**0.5160**	0.4807	0.3706	+0.0090
Conductance (LC)	Most-specific (baseline)	0.5067	0.4584	0.3537	–
	Mean over GO terms	0.5056	0.4625	0.3314	-0.0011
	Max over GO terms	0.5105	0.4899	0.3591	+0.0038
GradientSHAP (LGS)	Most-specific (baseline)	0.4925	0.4593	0.3368	–
	Mean over GO terms	0.4906	0.4206	0.3239	-0.0019
	Max over GO terms	0.5076	0.4552	0.3282	+0.0151

Table 10: Multi-GO-term attribution aggregation on the **ELMs** dataset. *Most-specific* is the original baseline, which attributes only the single most specific GO term; *Mean*/*Max* aggregate attributions over all positive GO terms (annotated term(s) plus all GO ancestors). ΔAUC is relative to that method's own baseline.

**Method**	**Aggregation**	**AUC**	**MRR**	**Top-30**	**ΔAUC**
Gradient×Activation (LGA)	Most-specific (baseline)	0.5925	0.1512	0.1683	–
	Mean over GO terms	**0.6291**	**0.1527**	**0.1800**	+0.0367
	Max over GO terms	0.6268	0.1417	0.1787	+0.0343
DeepLift (LDL)	Most-specific (baseline)	0.5908	0.1423	0.1673	–
	Mean over GO terms	0.6113	0.1428	0.1709	+0.0205
	Max over GO terms	0.6090	0.1400	0.1642	+0.0182
Integrated Gradients (LIG)	Most-specific (baseline)	0.5583	0.1205	0.1337	–
	Mean over GO terms	0.5667	0.0894	0.1259	+0.0084
	Max over GO terms	0.5570	0.0809	0.1202	-0.0013
Conductance (LC)	Most-specific (baseline)	0.5419	0.1215	0.1263	–
	Mean over GO terms	0.5524	0.1111	0.1345	+0.0105
	Max over GO terms	0.5459	0.1130	0.1275	+0.0040
GradientSHAP (LGS)	Most-specific (baseline)	0.5612	0.0989	0.1159	–
	Mean over GO terms	0.5924	0.1084	0.1473	+0.0312
	Max over GO terms	0.5776	0.1187	0.1281	+0.0164

Table 11: Multi-GO-term attribution aggregation on the **BioLIP** dataset. *Most-specific* is the original baseline, which attributes only the single most specific GO term; *Mean/Max* aggregate attributions over all positive GO terms (annotated term(s) plus all GO ancestors). ΔAUC is relative to that method's own baseline.

**Method**	**Aggregation**	**AUC**	**MRR**	**Top-30**	**ΔAUC**
Gradient×Activation (LGA)	Most-specific (baseline)	0.5974	0.1439	0.2374	–
	Mean over GO terms	0.6141	0.1490	0.2458	+0.0166
	Max over GO terms	0.5955	0.1391	0.2268	-0.0019
DeepLift (LDL)	Most-specific (baseline)	0.6160	0.1556	0.2460	–
	Mean over GO terms	**0.6265**	**0.1613**	**0.2524**	+0.0105
	Max over GO terms	0.6091	0.1437	0.2283	-0.0069
Integrated Gradients (LIG)	Most-specific (baseline)	0.5720	0.1176	0.1955	–
	Mean over GO terms	0.5823	0.1196	0.2078	+0.0103
	Max over GO terms	0.5727	0.1193	0.2059	+0.0008
Conductance (LC)	Most-specific (baseline)	0.6220	0.1122	0.1969	–
	Mean over GO terms	0.6244	0.1146	0.1900	+0.0024
	Max over GO terms	0.6228	0.1172	0.1915	+0.0008
GradientSHAP (LGS)	Most-specific (baseline)	0.5548	0.1086	0.1577	–
	Mean over GO terms	0.5755	0.1079	0.1818	+0.0207
	Max over GO terms	0.5522	0.1048	0.1640	-0.0026

Table 12: Multi-GO-term attribution aggregation on the **InterPro Domain** dataset. *Most-specific* is the original baseline, which attributes only the single most specific GO term; *Mean/Max* aggregate attributions over all positive GO terms (annotated term(s) plus all GO ancestors). ΔAUC is relative to that method's own baseline.

**Method**	**Aggregation**	**AUC**	**MRR**	**Top-30**	**ΔAUC**
Gradient×Activation (LGA)	Most-specific (baseline)	0.5545	0.5924	0.3518	–
	Mean over GO terms	**0.5727**	0.6358	0.3512	+0.0182
	Max over GO terms	0.5526	0.5639	0.3439	-0.0019
DeepLift (LDL)	Most-specific (baseline)	0.5496	0.6217	0.3603	–
	Mean over GO terms	0.5648	**0.6753**	**0.3604**	+0.0152
	Max over GO terms	0.5462	0.5995	0.3503	-0.0034
Integrated Gradients (LIG)	Most-specific (baseline)	0.5047	0.4720	0.3411	–
	Mean over GO terms	0.4917	0.5473	0.3219	-0.0130
	Max over GO terms	0.4919	0.5216	0.3283	-0.0128
Conductance (LC)	Most-specific (baseline)	0.4819	0.2952	0.2699	–
	Mean over GO terms	0.4788	0.2163	0.2392	-0.0031
	Max over GO terms	0.4724	0.2179	0.1941	-0.0096
GradientSHAP (LGS)	Most-specific (baseline)	0.4574	0.2732	0.2477	–
	Mean over GO terms	0.4450	0.1878	0.1895	-0.0125
	Max over GO terms	0.4529	0.2284	0.2426	-0.0045

Table 13: Multi-GO-term attribution aggregation on the **InterPro Active Site** dataset. *Most-specific* is the original baseline, which attributes only the single most specific GO term; *Mean/Max* aggregate attributions over all positive GO terms (annotated term(s) plus all GO ancestors). ΔAUC is relative to that method's own baseline.

**Method**	**Aggregation**	**AUC**	**MRR**	**Top-30**	**ΔAUC**
Gradient×Activation (LGA)	Most-specific (baseline)	0.5763	0.1826	0.1729	–
	Mean over GO terms	**0.5930**	0.1988	0.1885	+0.0167
	Max over GO terms	0.5805	0.1773	0.1693	+0.0041
DeepLift (LDL)	Most-specific (baseline)	0.5697	0.2061	0.1718	–
	Mean over GO terms	0.5802	**0.2296**	**0.1912**	+0.0106
	Max over GO terms	0.5695	0.2000	0.1695	-0.0002
Integrated Gradients (LIG)	Most-specific (baseline)	0.4763	0.1133	0.0885	–
	Mean over GO terms	0.4748	0.1225	0.0970	-0.0014
	Max over GO terms	0.4775	0.1219	0.1034	+0.0013
Conductance (LC)	Most-specific (baseline)	0.4614	0.0674	0.0753	–
	Mean over GO terms	0.4604	0.0665	0.0765	-0.0009
	Max over GO terms	0.4632	0.0775	0.0791	+0.0018
GradientSHAP (LGS)	Most-specific (baseline)	0.4859	0.0756	0.0778	–
	Mean over GO terms	0.4953	0.0768	0.0771	+0.0094
	Max over GO terms	0.4949	0.0785	0.0819	+0.0090

Table 14: Multi-GO-term attribution aggregation on the **InterPro Binding Site** dataset. *Most-specific* is the original baseline, which attributes only the single most specific GO term; *Mean/Max* aggregate attributions over all positive GO terms (annotated term(s) plus all GO ancestors). ΔAUC is relative to that method's own baseline.

**Method**	**Aggregation**	**AUC**	**MRR**	**Top-30**	**ΔAUC**
Gradient×Activation (LGA)	Most-specific (baseline)	0.5945	0.3580	0.1977	–
	Mean over GO terms	**0.6081**	0.4050	0.2089	+0.0136
	Max over GO terms	0.5894	0.4098	0.2017	-0.0051
DeepLift (LDL)	Most-specific (baseline)	0.5839	0.3589	0.1967	–
	Mean over GO terms	0.5961	**0.4118**	**0.2099**	+0.0123
	Max over GO terms	0.5801	0.4068	0.2027	-0.0037
Integrated Gradients (LIG)	Most-specific (baseline)	0.4995	0.1649	0.1355	–
	Mean over GO terms	0.5219	0.1857	0.1526	+0.0224
	Max over GO terms	0.5237	0.1767	0.1558	+0.0242
Conductance (LC)	Most-specific (baseline)	0.4960	0.1488	0.1398	–
	Mean over GO terms	0.4980	0.1484	0.1528	+0.0020
	Max over GO terms	0.4912	0.1485	0.1407	-0.0048
GradientSHAP (LGS)	Most-specific (baseline)	0.5043	0.1606	0.1421	–
	Mean over GO terms	0.5041	0.1660	0.1583	-0.0003
	Max over GO terms	0.5072	0.1619	0.1532	+0.0029

Table 15: Multi-GO-term attribution aggregation on the **InterPro Repeat** dataset. *Most-specific* is the original baseline, which attributes only the single most specific GO term; *Mean/Max* aggregate attributions over all positive GO terms (annotated term(s) plus all GO ancestors). ΔAUC is relative to that method's own baseline.

**Method**	**Aggregation**	**AUC**	**MRR**	**Top-30**	**ΔAUC**
Gradient×Activation (LGA)	Most-specific (baseline)	0.4749	0.2380	0.1279	–
	Mean over GO terms	0.4742	0.2122	0.1338	-0.0008
	Max over GO terms	0.4693	0.2385	0.1323	-0.0056
DeepLift (LDL)	Most-specific (baseline)	0.4841	0.2556	0.1343	–
	Mean over GO terms	0.4862	0.2295	0.1378	+0.0020
	Max over GO terms	0.4817	0.2539	0.1376	-0.0025
Integrated Gradients (LIG)	Most-specific (baseline)	0.5343	**0.2953**	**0.1823**	–
	Mean over GO terms	**0.5492**	0.2935	0.1815	+0.0150
	Max over GO terms	0.5479	0.2933	0.1769	+0.0136
Conductance (LC)	Most-specific (baseline)	0.4912	0.1948	0.1419	–
	Mean over GO terms	0.4884	0.1597	0.1399	-0.0027
	Max over GO terms	0.4886	0.1674	0.1452	-0.0026
GradientSHAP (LGS)	Most-specific (baseline)	0.5045	0.1705	0.1302	–
	Mean over GO terms	0.5025	0.1560	0.1353	-0.0019
	Max over GO terms	0.5017	0.1755	0.1400	-0.0027

Table 16: Multi-GO-term attribution aggregation on the **InterPro Homologous Superfamily** dataset. *Most-specific* is the original baseline, which attributes only the single most specific GO term; *Mean/Max* aggregate attributions over all positive GO terms (annotated term(s) plus all GO ancestors). ΔAUC is relative to that method's own baseline.

**Method**	**Aggregation**	**AUC**	**MRR**	**Top-30**	**ΔAUC**
Gradient×Activation (LGA)	Most-specific (baseline)	0.4565	0.4751	0.2486	–
	Mean over GO terms	0.4539	0.4332	0.2332	-0.0026
	Max over GO terms	0.4573	0.4287	0.2414	+0.0008
DeepLift (LDL)	Most-specific (baseline)	0.4651	0.4676	0.2549	–
	Mean over GO terms	0.4662	0.4319	0.2348	+0.0011
	Max over GO terms	0.4694	0.4374	0.2396	+0.0043
Integrated Gradients (LIG)	Most-specific (baseline)	0.4861	**0.4992**	0.2750	–
	Mean over GO terms	0.4605	0.4672	0.2686	-0.0257
	Max over GO terms	0.4650	0.4666	**0.2857**	-0.0212
Conductance (LC)	Most-specific (baseline)	0.4952	0.3454	0.2569	–
	Mean over GO terms	0.4971	0.3390	0.2514	+0.0019
	Max over GO terms	**0.4971**	0.3650	0.2581	+0.0019
GradientSHAP (LGS)	Most-specific (baseline)	0.4723	0.2859	0.2451	–
	Mean over GO terms	0.4790	0.2508	0.2479	+0.0068
	Max over GO terms	0.4857	0.2974	0.2747	+0.0135

Table 17: Residue-level metrics on **CSA**, stratified by the GO depth of `GOTerm[0]` (split at the dataset median). All proteins: n=784; Low depth: n=532; High depth: n=252. ΔAUC is high minus low.

**Method**	**All proteins**			**Low depth**			**High depth**		
	AUC	*MRR*	*Top-30*	*AUC*	*MRR*	*Top-30*	*AUC*	*MRR*	*T*
Masked Entropy	0.8158	0.4038	0.5945	0.8135	0.4012	0.5810	0.8206	0.4092	0
Masked Entropy (Cond)	0.8113	0.3403	0.5811	0.8082	0.3458	0.5665	0.8179	0.3287	0
Masked Entropy (Cond Span)	0.7412	0.2845	0.4069	0.7385	0.2949	0.4039	0.7469	0.2626	0
Entropy Gain (Cond)	0.2763	0.0383	0.0527	0.2799	0.0393	0.0560	0.2685	0.0360	0
Entropy Gain (Cond Span)	0.4970	0.0950	0.1234	0.4998	0.0988	0.1292	0.4911	0.0870	0
LGA Attribution	0.6044	0.1143	0.1723	0.6130	0.1240	0.1761	0.5864	0.0939	0
LDL Attribution	0.6206	0.1177	0.1873	0.6305	0.1255	0.1950	0.5998	0.1011	0
LGS Attribution	0.5393	0.0645	0.1079	0.5453	0.0685	0.1191	0.5267	0.0560	0
LIG Attribution	0.5449	0.0628	0.1114	0.5562	0.0650	0.1175	0.5211	0.0581	0
LC Attribution	0.6035	0.0667	0.1210	0.6159	0.0711	0.1287	0.5774	0.0573	0
LGA Attribution (GO-mean)	0.6388	0.1393	0.1926	0.6525	0.1473	0.1922	0.6097	0.1226	0
LDL Attribution (GO-mean)	0.6496	0.1437	0.2040	0.6622	0.1519	0.2077	0.6230	0.1263	0
Attention Attribution	0.5866	0.0804	0.1311	0.5742	0.0775	0.1154	0.6128	0.0864	0
Naive	0.5063	0.0604	0.0986	0.5052	0.0599	0.0977	0.5086	0.0614	0
LGA Attribution (GO-max)	0.6154	0.1322	0.1723	0.6307	0.1443	0.1801	0.5830	0.1066	0
LGS Attribution (GO-mean)	0.5424	0.0653	0.1144	0.5517	0.0666	0.1215	0.5230	0.0625	0
Attention (layer 0)	0.6208	0.0800	0.1380	0.6236	0.0782	0.1396	0.6148	0.0839	0
LIG Attribution (GO-mean)	0.5504	0.0640	0.1253	0.5602	0.0654	0.1279	0.5299	0.0610	0
LIG Attribution (GO-sum)	0.5504	0.0640	0.1253	0.5602	0.0654	0.1279	0.5299	0.0610	0
LDL Attribution (GO-max)	0.6263	0.1346	0.1873	0.6410	0.1439	0.1980	0.5952	0.1148	0
Masked Entropy (Cond Span, 100/5)	0.7303	0.2579	0.3774	0.7238	0.2619	0.3660	0.7440	0.2494	0
Attention (layer 35)	0.3587	0.0873	0.1029	0.3683	0.0916	0.1119	0.3384	0.0782	0
LGS Attribution (GO-sum)	0.5424	0.0653	0.1144	0.5517	0.0666	0.1215	0.5230	0.0625	0
LC Attribution (GO-mean)	0.6021	0.0678	0.1257	0.6142	0.0738	0.1319	0.5764	0.0550	0
LIG Attribution (most-specific)	0.5449	0.0628	0.1114	0.5562	0.0650	0.1175	0.5211	0.0581	0
Masked Entropy (Cond Span, 200/5)	0.7377	0.2602	0.3866	0.7342	0.2685	0.3880	0.7451	0.2428	0
Attention (layer 9)	0.4950	0.0546	0.0650	0.5017	0.0604	0.0656	0.4807	0.0424	0
LC Attribution (most-specific)	0.6035	0.0667	0.1210	0.6159	0.0711	0.1287	0.5774	0.0573	0
Masked Entropy (Cond Span, 100/2)	0.7500	0.3190	0.4347	0.7442	0.3155	0.4180	0.7621	0.3265	0
LC Attribution (GO-max)	0.6045	0.0756	0.1273	0.6153	0.0835	0.1277	0.5816	0.0589	0
Masked Entropy (Cond Span, 50/5)	0.6922	0.2499	0.3209	0.6820	0.2583	0.3167	0.7138	0.2322	0
LGS Attribution (GO-max)	0.5323	0.0629	0.1056	0.5384	0.0629	0.1144	0.5193	0.0628	0
LGA Attribution (GO-sum)	0.6388	0.1393	0.1926	0.6525	0.1473	0.1922	0.6097	0.1226	0
LC Attribution (GO-sum)	0.6021	0.0678	0.1257	0.6142	0.0738	0.1319	0.5764	0.0550	0
Masked Entropy (Cond Span, 100/10)	0.7035	0.2327	0.3048	0.6999	0.2368	0.2987	0.7113	0.2241	0
LDL Attribution (GO-sum)	0.6496	0.1437	0.2040	0.6622	0.1519	0.2077	0.6230	0.1263	0
IG Attribution (most-specific)	0.4779	0.0523	0.0843	0.4850	0.0500	0.0869	0.4629	0.0570	0
LIG Attribution (GO-max)	0.5460	0.0659	0.1235	0.5553	0.0684	0.1267	0.5265	0.0604	0
Attention (layer 18)	0.6399	0.0763	0.1619	0.6429	0.0706	0.1613	0.6338	0.0883	0
LDL Attribution (most-specific)	0.6206	0.1177	0.1873	0.6305	0.1255	0.1950	0.5998	0.1011	0
LGA Attribution (most-specific)	0.6044	0.1143	0.1723	0.6130	0.1240	0.1761	0.5864	0.0939	0
LGS Attribution (most-specific)	0.5448	0.0597	0.0950	0.5490	0.0627	0.1037	0.5359	0.0532	0

Table 18: Residue-level metrics on **LLPS**, stratified by the GO depth of `GOTerm[0]` (split at the dataset median). All proteins: n=53; Low depth: n=28; High depth: n=25. ΔAUC is high minus low.

**Method**	**All proteins**			**Low depth**			**High depth**		
	AUC	*MRR*	*Top-30*	*AUC*	*MRR*	*Top-30*	*AUC*	*MRR*	*T*
Masked Entropy	0.4498	0.3268	0.3036	0.4481	0.2910	0.2983	0.4517	0.3668	0
Masked Entropy (Cond)	0.4491	0.3101	0.2813	0.4387	0.3049	0.2583	0.4608	0.3160	0
Masked Entropy (Cond Span)	0.4743	0.3001	0.3063	0.4757	0.3537	0.2726	0.4728	0.2401	0
Entropy Gain (Cond)	0.5067	0.5283	0.3534	0.5112	0.5695	0.3528	0.5016	0.4822	0
Entropy Gain (Cond Span)	0.5007	0.4094	0.3143	0.5218	0.4693	0.3097	0.4770	0.3423	0
LGA Attribution	0.4898	0.4520	0.3377	0.5075	0.4710	0.3306	0.4698	0.4307	0
LDL Attribution	0.4897	0.3801	0.3244	0.5016	0.3852	0.3075	0.4764	0.3744	0
LGS Attribution	0.5047	0.4358	0.3528	0.5067	0.4742	0.3721	0.5025	0.3927	0
LIG Attribution	0.5069	0.5008	0.3857	0.5060	0.4184	0.3762	0.5080	0.5930	0
LC Attribution	0.5067	0.4584	0.3537	0.5156	0.4755	0.3655	0.4968	0.4394	0
LGA Attribution (GO-mean)	0.4868	0.4620	0.3224	0.4934	0.3996	0.3007	0.4793	0.5319	0
LDL Attribution (GO-mean)	0.4882	0.4529	0.3131	0.4901	0.4284	0.2852	0.4862	0.4804	0
Attention Attribution	0.4508	0.4256	0.2760	0.4199	0.3792	0.2543	0.4854	0.4775	0
Naive	0.5016	0.6096	0.3511	0.5028	0.5965	0.3512	0.5003	0.6243	0
LGA Attribution (GO-max)	0.4910	0.4498	0.3235	0.4969	0.4227	0.2881	0.4844	0.4802	0
LGS Attribution (GO-mean)	0.4906	0.4206	0.3239	0.4992	0.4769	0.3519	0.4810	0.3576	0
Attention (layer 0)	0.5061	0.4519	0.4280	0.5055	0.3989	0.4036	0.5067	0.5112	0
LIG Attribution (GO-mean)	0.5151	0.4788	0.3719	0.5195	0.4344	0.3857	0.5101	0.5284	0
LIG Attribution (GO-sum)	0.5151	0.4788	0.3719	0.5195	0.4344	0.3857	0.5101	0.5284	0
LDL Attribution (GO-max)	0.4916	0.4263	0.3084	0.4946	0.3918	0.2810	0.4883	0.4649	0
Masked Entropy (Cond Span, 100/5)	0.4701	0.3051	0.2931	0.4685	0.3192	0.2452	0.4718	0.2892	0
Attention (layer 35)	0.4844	0.4683	0.3262	0.4741	0.4738	0.3209	0.4960	0.4623	0
LGS Attribution (GO-sum)	0.4906	0.4206	0.3239	0.4992	0.4769	0.3519	0.4810	0.3576	0
LC Attribution (GO-mean)	0.5056	0.4625	0.3314	0.5137	0.5215	0.3447	0.4965	0.3964	0
LIG Attribution (most-specific)	0.5069	0.5008	0.3857	0.5060	0.4184	0.3762	0.5080	0.5930	0
Masked Entropy (Cond Span, 200/5)	0.4608	0.3282	0.2918	0.4493	0.3594	0.2690	0.4737	0.2932	0
Attention (layer 9)	0.3826	0.3503	0.2847	0.3807	0.3264	0.2626	0.3847	0.3771	0
LC Attribution (most-specific)	0.5067	0.4584	0.3537	0.5156	0.4755	0.3655	0.4968	0.4394	0
Masked Entropy (Cond Span, 100/2)	0.4775	0.3021	0.2937	0.4759	0.3013	0.2548	0.4793	0.3030	0
LC Attribution (GO-max)	0.5105	0.4899	0.3591	0.5135	0.5175	0.3435	0.5071	0.4590	0
Masked Entropy (Cond Span, 50/5)	0.5120	0.3344	0.3189	0.5231	0.3185	0.2929	0.4996	0.3523	0
LGS Attribution (GO-max)	0.5076	0.4552	0.3282	0.5055	0.5056	0.3555	0.5099	0.3987	0
LGA Attribution (GO-sum)	0.4868	0.4620	0.3224	0.4934	0.3996	0.3007	0.4793	0.5319	0
LC Attribution (GO-sum)	0.5056	0.4625	0.3314	0.5137	0.5215	0.3447	0.4965	0.3964	0
Masked Entropy (Cond Span, 100/10)	0.4887	0.3299	0.2994	0.4956	0.3372	0.2583	0.4810	0.3216	0
LDL Attribution (GO-sum)	0.4882	0.4529	0.3131	0.4901	0.4284	0.2852	0.4862	0.4804	0
IG Attribution (most-specific)	0.4794	0.5267	0.3409	0.5102	0.4475	0.3271	0.4450	0.6153	0
LIG Attribution (GO-max)	0.5160	0.4807	0.3706	0.5214	0.4216	0.3750	0.5098	0.5468	0
Attention (layer 18)	0.4821	0.3857	0.3802	0.4822	0.3810	0.3857	0.4819	0.3909	0
LDL Attribution (most-specific)	0.4897	0.3801	0.3244	0.5016	0.3852	0.3075	0.4764	0.3744	0
LGA Attribution (most-specific)	0.4898	0.4520	0.3377	0.5075	0.4710	0.3306	0.4698	0.4307	0
LGS Attribution (most-specific)	0.4925	0.4593	0.3368	0.5053	0.4634	0.3762	0.4782	0.4548	0

Table 19: Residue-level metrics on **ELMs**, stratified by the GO depth of `GOTerm[0]` (split at the dataset median). All proteins: n=229; Low depth: n=104; High depth: n=101. ΔAUC is high minus low.

**Method**	**All proteins**			**Low depth**			**High depth**		
	AUC	*MRR*	*Top-30*	*AUC*	*MRR*	*Top-30*	*AUC*	*MRR*	*T*
Masked Entropy	0.5210	0.0766	0.0901	0.4986	0.0689	0.0817	0.5283	0.0854	0
Masked Entropy (Cond)	0.5092	0.0744	0.0869	0.4857	0.0715	0.0765	0.5252	0.0764	0
Masked Entropy (Cond Span)	0.3909	0.0596	0.0562	0.4190	0.0622	0.0486	0.3613	0.0581	0
Entropy Gain (Cond)	0.5280	0.1273	0.1261	0.5242	0.1151	0.1077	0.5352	0.1490	0
Entropy Gain (Cond Span)	0.5083	0.0906	0.0728	0.4947	0.0908	0.0536	0.5210	0.0893	0
LGA Attribution	0.5925	0.1512	0.1683	0.5974	0.1444	0.1590	0.5940	0.1700	0
LDL Attribution	0.5908	0.1423	0.1673	0.5959	0.1330	0.1564	0.5962	0.1649	0
LGS Attribution	0.5498	0.0925	0.1087	0.5626	0.0951	0.1069	0.5397	0.0903	0
LIG Attribution	0.5583	0.1205	0.1337	0.5521	0.1099	0.1134	0.5558	0.1465	0
LGA Attribution (GO-mean)	0.6291	0.1527	0.1800	0.6333	0.1519	0.1765	0.6276	0.1704	0
LDL Attribution (GO-mean)	0.6113	0.1428	0.1709	0.6102	0.1245	0.1727	0.6181	0.1737	0
Attention Attribution	0.4880	0.1138	0.0929	0.4743	0.0947	0.0879	0.4874	0.1091	0
Naive	0.4977	0.0924	0.0915	0.4935	0.0865	0.0869	0.5113	0.0894	0
LGA Attribution (GO-max)	0.6268	0.1417	0.1787	0.6260	0.1200	0.1736	0.6267	0.1824	0
LGS Attribution (GO-mean)	0.5924	0.1084	0.1473	0.6026	0.1108	0.1288	0.5779	0.1156	0
Attention (layer 0)	0.4442	0.1049	0.1007	0.4493	0.1155	0.1079	0.4368	0.0981	0
LIG Attribution (GO-mean)	0.5667	0.0894	0.1259	0.5629	0.0739	0.1113	0.5705	0.1166	0
LIG Attribution (GO-sum)	0.5667	0.0894	0.1259	0.5629	0.0739	0.1113	0.5705	0.1166	0
LDL Attribution (GO-max)	0.6090	0.1400	0.1642	0.6011	0.1210	0.1607	0.6173	0.1778	0
Masked Entropy (Cond Span, 100/5)	0.3851	0.0450	0.0548	0.4122	0.0526	0.0440	0.3647	0.0400	0
Attention (layer 35)	0.5130	0.1019	0.0934	0.4923	0.0834	0.0737	0.5252	0.1116	0
LGS Attribution (GO-sum)	0.5924	0.1084	0.1473	0.6026	0.1108	0.1288	0.5779	0.1156	0
LC Attribution (GO-mean)	0.5524	0.1111	0.1345	0.5572	0.1207	0.1233	0.5521	0.0980	0
LIG Attribution (most-specific)	0.5583	0.1205	0.1337	0.5521	0.1099	0.1134	0.5558	0.1465	0
Masked Entropy (Cond Span, 200/5)	0.3768	0.0508	0.0548	0.4178	0.0660	0.0684	0.3510	0.0404	0
Attention (layer 9)	0.4559	0.0781	0.0759	0.4594	0.0721	0.0636	0.4406	0.0755	0
LC Attribution (most-specific)	0.5419	0.1215	0.1263	0.5484	0.1222	0.1312	0.5458	0.1174	0
Masked Entropy (Cond Span, 100/2)	0.3969	0.0526	0.0528	0.4188	0.0659	0.0468	0.3840	0.0363	0
LC Attribution (GO-max)	0.5459	0.1130	0.1275	0.5518	0.1161	0.1231	0.5472	0.1031	0
Masked Entropy (Cond Span, 50/5)	0.4142	0.0487	0.0531	0.4188	0.0508	0.0429	0.4018	0.0439	0
LGS Attribution (GO-max)	0.5776	0.1187	0.1281	0.5840	0.1130	0.1102	0.5686	0.1272	0
LGA Attribution (GO-sum)	0.6291	0.1527	0.1800	0.6333	0.1519	0.1765	0.6276	0.1704	0
LC Attribution (GO-sum)	0.5524	0.1111	0.1345	0.5572	0.1207	0.1233	0.5521	0.0980	0
Masked Entropy (Cond Span, 100/10)	0.3800	0.0432	0.0458	0.4090	0.0541	0.0428	0.3570	0.0284	0
LDL Attribution (GO-sum)	0.6113	0.1428	0.1709	0.6102	0.1245	0.1727	0.6181	0.1737	0
IG Attribution (most-specific)	0.5455	0.0810	0.0899	0.5627	0.0762	0.0889	0.5295	0.0792	0
LIG Attribution (GO-max)	0.5570	0.0809	0.1202	0.5534	0.0760	0.1103	0.5598	0.0948	0
Attention (layer 18)	0.5181	0.1141	0.1256	0.5138	0.0775	0.1183	0.4981	0.1437	0
LDL Attribution (most-specific)	0.5908	0.1423	0.1673	0.5959	0.1330	0.1564	0.5962	0.1649	0
LGA Attribution (most-specific)	0.5925	0.1512	0.1683	0.5974	0.1444	0.1590	0.5940	0.1700	0
LGS Attribution (most-specific)	0.5612	0.0989	0.1159	0.5629	0.0785	0.0917	0.5556	0.1342	0

Table 20: Residue-level metrics on **BioLiP**, stratified by the GO depth of `GOTerm[0]` (split at the dataset median). All proteins: n=758; Low depth: n=470; High depth: n=286. ΔAUC is high minus low.

**Method**	**All proteins**			**Low depth**			**High dept**		
	AUC	*MRR*	*Top-30*	*AUC*	*MRR*	*Top-30*	*AUC*	*MRR*	*T*
Masked Entropy	0.7287	0.3404	0.4713	0.7273	0.3535	0.4842	0.7327	0.3208	0
Masked Entropy (Cond)	0.7224	0.3131	0.4558	0.7220	0.3414	0.4725	0.7250	0.2682	0
Masked Entropy (Cond Span)	0.6686	0.2672	0.3669	0.6677	0.2859	0.3743	0.6710	0.2377	0
Entropy Gain (Cond)	0.3674	0.1066	0.1176	0.3669	0.1228	0.1274	0.3671	0.0799	0
Entropy Gain (Cond Span)	0.5011	0.1228	0.1675	0.4946	0.1260	0.1714	0.5132	0.1180	0
LGA Attribution	0.5974	0.1439	0.2374	0.6151	0.1722	0.2690	0.5686	0.0973	0
LDL Attribution	0.6160	0.1556	0.2460	0.6338	0.1879	0.2799	0.5872	0.1016	0
LGS Attribution	0.5673	0.1107	0.1744	0.5731	0.1240	0.1806	0.5582	0.0860	0
LIG Attribution	0.5720	0.1176	0.1955	0.5788	0.1261	0.2095	0.5612	0.1033	0
LGA Attribution (GO-mean)	0.6141	0.1490	0.2458	0.6324	0.1775	0.2741	0.5845	0.1017	0
LDL Attribution (GO-mean)	0.6265	0.1613	0.2524	0.6447	0.1892	0.2848	0.5974	0.1150	0
Attention Attribution	0.5695	0.1199	0.2004	0.5791	0.1311	0.2135	0.5539	0.1021	0
Naive	0.5111	0.1140	0.1427	0.5045	0.1173	0.1485	0.5216	0.1072	0
LGA Attribution (GO-max)	0.5955	0.1391	0.2268	0.6124	0.1613	0.2598	0.5688	0.1023	0
LGS Attribution (GO-mean)	0.5755	0.1079	0.1818	0.5836	0.1247	0.2002	0.5627	0.0804	0
Attention (layer 0)	0.5968	0.1327	0.1989	0.6033	0.1335	0.2026	0.5858	0.1288	0
LIG Attribution (GO-mean)	0.5823	0.1196	0.2078	0.5869	0.1235	0.2107	0.5753	0.1128	0
LIG Attribution (GO-sum)	0.5823	0.1196	0.2078	0.5869	0.1235	0.2107	0.5753	0.1128	0
LDL Attribution (GO-max)	0.6091	0.1437	0.2283	0.6260	0.1630	0.2577	0.5825	0.1109	0
Masked Entropy (Cond Span, 100/5)	0.6610	0.2462	0.3420	0.6566	0.2741	0.3473	0.6688	0.2015	0
Attention (layer 35)	0.3948	0.1376	0.1324	0.3952	0.1529	0.1457	0.3928	0.1090	0
LGS Attribution (GO-sum)	0.5755	0.1079	0.1818	0.5836	0.1247	0.2002	0.5627	0.0804	0
LC Attribution (GO-mean)	0.6244	0.1146	0.1900	0.6296	0.1244	0.2055	0.6170	0.0987	0
LIG Attribution (most-specific)	0.5720	0.1176	0.1955	0.5788	0.1261	0.2095	0.5612	0.1033	0
Masked Entropy (Cond Span, 200/5)	0.6631	0.2332	0.3283	0.6541	0.2514	0.3340	0.6786	0.2044	0
Attention (layer 9)	0.4717	0.0859	0.1218	0.4640	0.0910	0.1185	0.4850	0.0762	0
LC Attribution (most-specific)	0.6220	0.1122	0.1969	0.6249	0.1181	0.2108	0.6183	0.1032	0
Masked Entropy (Cond Span, 100/2)	0.6690	0.2834	0.3705	0.6673	0.3110	0.3809	0.6729	0.2393	0
LC Attribution (GO-max)	0.6228	0.1172	0.1915	0.6272	0.1202	0.2059	0.6167	0.1128	0
Masked Entropy (Cond Span, 50/5)	0.6346	0.2659	0.3227	0.6293	0.2779	0.3297	0.6451	0.2480	0
LGS Attribution (GO-max)	0.5522	0.1048	0.1640	0.5587	0.1173	0.1810	0.5417	0.0825	0
LGA Attribution (GO-sum)	0.6141	0.1490	0.2458	0.6324	0.1775	0.2741	0.5845	0.1017	0
LC Attribution (GO-sum)	0.6244	0.1146	0.1900	0.6296	0.1244	0.2055	0.6170	0.0987	0
Masked Entropy (Cond Span, 100/10)	0.6439	0.2212	0.3166	0.6429	0.2458	0.3253	0.6462	0.1817	0
LDL Attribution (GO-sum)	0.6265	0.1613	0.2524	0.6447	0.1892	0.2848	0.5974	0.1150	0
LIG Attribution (GO-max)	0.5727	0.1193	0.2059	0.5793	0.1217	0.2098	0.5624	0.1149	0
Attention (layer 18)	0.6106	0.1211	0.2299	0.6182	0.1300	0.2385	0.5994	0.1070	0
LDL Attribution (most-specific)	0.6160	0.1556	0.2460	0.6338	0.1879	0.2799	0.5872	0.1016	0
LGA Attribution (most-specific)	0.5974	0.1439	0.2374	0.6151	0.1722	0.2690	0.5686	0.0973	0
LGS Attribution (most-specific)	0.5548	0.1086	0.1577	0.5657	0.1177	0.1731	0.5372	0.0935	0

Table 21: Residue-level metrics on **Repeat**, stratified by the GO depth of `GOTerm[0]` (split at the dataset median). All proteins: n=257; Low depth: n=151; High depth: n=106. ΔAUC is high minus low.

**Method**	**All proteins**			**Low depth**			**High depth**		
	AUC	*MRR*	*Top-30*	*AUC*	*MRR*	*Top-30*	*AUC*	*MRR*	*T*
Masked Entropy	0.6179	0.3710	0.2284	0.6045	0.3468	0.1972	0.6369	0.4055	0
Masked Entropy (Cond)	0.6273	0.3549	0.2340	0.6123	0.2579	0.1993	0.6486	0.4930	0
Masked Entropy (Cond Span)	0.6661	0.3463	0.2671	0.6493	0.3248	0.2330	0.6900	0.3769	0
Entropy Gain (Cond)	0.5313	0.2891	0.1616	0.5412	0.2689	0.1506	0.5172	0.3180	0
Entropy Gain (Cond Span)	0.4770	0.3341	0.1935	0.4760	0.2892	0.1803	0.4785	0.3981	0
LGA Attribution	0.4749	0.2380	0.1279	0.4949	0.2282	0.1369	0.4465	0.2520	0
LDL Attribution	0.4841	0.2556	0.1343	0.5045	0.2487	0.1451	0.4552	0.2654	0
LGS Attribution	0.5071	0.1612	0.1361	0.5199	0.1636	0.1313	0.4887	0.1577	0
LIG Attribution	0.5343	0.2953	0.1823	0.5269	0.2795	0.1775	0.5449	0.3179	0
LGA Attribution (GO-mean)	0.4742	0.2122	0.1338	0.5017	0.2251	0.1433	0.4349	0.1937	0
LDL Attribution (GO-mean)	0.4862	0.2295	0.1378	0.5109	0.2404	0.1469	0.4510	0.2140	0
Attention Attribution	0.5875	0.4079	0.1879	0.5857	0.3574	0.1672	0.5900	0.4798	0
Naive	0.4962	0.3165	0.1483	0.5006	0.2814	0.1367	0.4899	0.3665	0
LGA Attribution (GO-max)	0.4693	0.2385	0.1323	0.4955	0.2324	0.1418	0.4319	0.2473	0
LGS Attribution (GO-mean)	0.5025	0.1560	0.1353	0.5071	0.1547	0.1296	0.4960	0.1577	0
Attention (layer 0)	0.4748	0.2123	0.1144	0.4627	0.2434	0.1174	0.4920	0.1680	0
LIG Attribution (GO-mean)	0.5492	0.2935	0.1815	0.5431	0.2988	0.1740	0.5579	0.2860	0
LIG Attribution (GO-sum)	0.5492	0.2935	0.1815	0.5431	0.2988	0.1740	0.5579	0.2860	0
LDL Attribution (GO-max)	0.4817	0.2539	0.1376	0.5051	0.2557	0.1446	0.4483	0.2513	0
Masked Entropy (Cond Span, 100/5)	0.6643	0.3574	0.2610	0.6491	0.3096	0.2261	0.6859	0.4254	0
Attention (layer 35)	0.5582	0.3429	0.1818	0.5640	0.2972	0.1659	0.5499	0.4079	0
LGS Attribution (GO-sum)	0.5025	0.1560	0.1353	0.5071	0.1547	0.1296	0.4960	0.1577	0
LC Attribution (GO-mean)	0.4884	0.1597	0.1399	0.4937	0.1723	0.1400	0.4809	0.1418	0
LIG Attribution (most-specific)	0.5343	0.2953	0.1823	0.5269	0.2795	0.1775	0.5449	0.3179	0
Masked Entropy (Cond Span, 200/5)	0.6490	0.3077	0.2262	0.6215	0.2367	0.1624	0.6882	0.4088	0
Attention (layer 9)	0.5249	0.3619	0.1726	0.5312	0.3832	0.1496	0.5160	0.3316	0
LC Attribution (most-specific)	0.4912	0.1948	0.1419	0.4981	0.2073	0.1490	0.4814	0.1771	0
Masked Entropy (Cond Span, 100/2)	0.6701	0.4579	0.2691	0.6556	0.3790	0.2347	0.6907	0.5703	0
LC Attribution (GO-max)	0.4886	0.1674	0.1452	0.4934	0.1814	0.1470	0.4818	0.1474	0
Masked Entropy (Cond Span, 50/5)	0.6814	0.4527	0.2858	0.6638	0.3697	0.2300	0.7065	0.5708	0
LGS Attribution (GO-max)	0.5017	0.1755	0.1400	0.5068	0.1740	0.1360	0.4945	0.1776	0
LGA Attribution (GO-sum)	0.4742	0.2122	0.1338	0.5017	0.2251	0.1433	0.4349	0.1937	0
LC Attribution (GO-sum)	0.4884	0.1597	0.1399	0.4937	0.1723	0.1400	0.4809	0.1418	0
Masked Entropy (Cond Span, 100/10)	0.6584	0.3755	0.2591	0.6400	0.3266	0.2262	0.6846	0.4451	0
LDL Attribution (GO-sum)	0.4862	0.2295	0.1378	0.5109	0.2404	0.1469	0.4510	0.2140	0
LIG Attribution (GO-max)	0.5479	0.2933	0.1769	0.5426	0.2939	0.1719	0.5555	0.2925	0
Attention (layer 18)	0.5419	0.4240	0.1947	0.5484	0.3775	0.1794	0.5326	0.4903	0
LDL Attribution (most-specific)	0.4841	0.2556	0.1343	0.5045	0.2487	0.1451	0.4552	0.2654	0
LGA Attribution (most-specific)	0.4749	0.2380	0.1279	0.4949	0.2282	0.1369	0.4465	0.2520	0
LGS Attribution (most-specific)	0.5045	0.1705	0.1302	0.5082	0.1776	0.1273	0.4991	0.1605	0

Table 22: Residue-level metrics on **Domain**, stratified by the GO depth of `GOTerm[0]` (split at the dataset median). All proteins: n=179; Low depth: n=102; High depth: n=77. ΔAUC is high minus low.

**Method**	**All proteins**			**Low depth**			**High depth**		
	AUC	*MRR*	*Top-30*	*AUC*	*MRR*	*Top-30*	*AUC*	*MRR*	*T*
Masked Entropy	0.7856	0.7868	0.7002	0.8227	0.8872	0.8061	0.7366	0.6538	0
Masked Entropy (Cond)	0.7972	0.7031	0.6840	0.8263	0.7194	0.7652	0.7587	0.6814	0
Masked Entropy (Cond Span)	0.8278	0.6775	0.7041	0.8742	0.7907	0.8280	0.7664	0.5276	0
Entropy Gain (Cond)	0.4774	0.4649	0.3513	0.4656	0.4901	0.3757	0.4931	0.4316	0
Entropy Gain (Cond Span)	0.5064	0.7077	0.6216	0.4988	0.8022	0.7329	0.5164	0.5824	0
LGA Attribution	0.5545	0.5924	0.3518	0.5231	0.5439	0.3622	0.5961	0.6565	0
LDL Attribution	0.5496	0.6217	0.3603	0.5241	0.6057	0.3755	0.5835	0.6430	0
LGS Attribution	0.4620	0.2475	0.2317	0.4576	0.2893	0.2734	0.4677	0.1921	0
LIG Attribution	0.5047	0.4720	0.3411	0.5365	0.5498	0.4125	0.4627	0.3689	0
LGA Attribution (GO-mean)	0.5727	0.6358	0.3512	0.5409	0.6053	0.3666	0.6149	0.6761	0
LDL Attribution (GO-mean)	0.5648	0.6753	0.3604	0.5388	0.6617	0.3773	0.5993	0.6932	0
Attention Attribution	0.6239	0.7991	0.4603	0.6069	0.8172	0.5216	0.6464	0.7752	0
Naive	0.4976	0.4911	0.3261	0.4947	0.5482	0.3649	0.5014	0.4154	0
LGA Attribution (GO-max)	0.5526	0.5639	0.3439	0.5159	0.4898	0.3646	0.6012	0.6621	0
LGS Attribution (GO-mean)	0.4450	0.1878	0.1895	0.4455	0.1888	0.2127	0.4442	0.1866	0
Attention (layer 0)	0.5056	0.3083	0.2783	0.5031	0.2346	0.3234	0.5088	0.4059	0
LIG Attribution (GO-mean)	0.4917	0.5473	0.3219	0.5190	0.6660	0.3825	0.4556	0.3902	0
LIG Attribution (GO-sum)	0.4917	0.5473	0.3219	0.5190	0.6660	0.3825	0.4556	0.3902	0
LDL Attribution (GO-max)	0.5462	0.5995	0.3503	0.5189	0.5422	0.3751	0.5824	0.6753	0
Masked Entropy (Cond Span, 100/5)	0.8294	0.6870	0.7103	0.8865	0.8196	0.8395	0.7537	0.5115	0
Attention (layer 35)	0.5068	0.5085	0.4570	0.4969	0.5720	0.5526	0.5200	0.4243	0
LGS Attribution (GO-sum)	0.4450	0.1878	0.1895	0.4455	0.1888	0.2127	0.4442	0.1866	0
LC Attribution (GO-mean)	0.4788	0.2163	0.2392	0.4599	0.2565	0.2577	0.5038	0.1630	0
LIG Attribution (most-specific)	0.5047	0.4720	0.3411	0.5365	0.5498	0.4125	0.4627	0.3689	0
Masked Entropy (Cond Span, 200/5)	0.8571	0.7230	0.7337	0.8987	0.7793	0.8216	0.8018	0.6485	0
Attention (layer 9)	0.6277	0.7816	0.4248	0.6289	0.9678	0.4696	0.6261	0.5349	0
LC Attribution (most-specific)	0.4819	0.2952	0.2699	0.4659	0.3724	0.2890	0.5031	0.1929	0
Masked Entropy (Cond Span, 100/2)	0.8323	0.8237	0.7252	0.8841	0.8684	0.8403	0.7636	0.7645	0
LC Attribution (GO-max)	0.4724	0.2179	0.1941	0.4490	0.2570	0.1718	0.5034	0.1660	0
Masked Entropy (Cond Span, 50/5)	0.8357	0.7903	0.7546	0.8837	0.9039	0.8964	0.7722	0.6397	0
LGS Attribution (GO-max)	0.4529	0.2284	0.2426	0.4578	0.2537	0.2779	0.4465	0.1949	0
LGA Attribution (GO-sum)	0.5727	0.6358	0.3512	0.5409	0.6053	0.3666	0.6149	0.6761	0
LC Attribution (GO-sum)	0.4788	0.2163	0.2392	0.4599	0.2565	0.2577	0.5038	0.1630	0
Masked Entropy (Cond Span, 100/10)	0.8492	0.7165	0.7293	0.9115	0.8150	0.8719	0.7667	0.5861	0
LDL Attribution (GO-sum)	0.5648	0.6753	0.3604	0.5388	0.6617	0.3773	0.5993	0.6932	0
LIG Attribution (GO-max)	0.4919	0.5216	0.3283	0.5162	0.6180	0.3967	0.4597	0.3939	0
Attention (layer 18)	0.5804	0.8465	0.3061	0.5811	0.8827	0.3238	0.5795	0.7986	0
LDL Attribution (most-specific)	0.5496	0.6217	0.3603	0.5241	0.6057	0.3755	0.5835	0.6430	0
LGA Attribution (most-specific)	0.5545	0.5924	0.3518	0.5231	0.5439	0.3622	0.5961	0.6565	0
LGS Attribution (most-specific)	0.4574	0.2732	0.2477	0.4594	0.2958	0.2877	0.4548	0.2434	0

Table 23: Residue-level metrics on **Binding Site**, stratified by the GO depth of `GOTerm[0]` (split at the dataset median). All proteins: n=462; Low depth: n=265; High depth: n=197. ΔAUC is high minus low.

**Method**	**All proteins**			**Low depth**			**High depth**		
	AUC	*MRR*	*Top-30*	*AUC*	*MRR*	*Top-30*	*AUC*	*MRR*	*T*
Masked Entropy	0.7160	0.5162	0.3226	0.7096	0.4481	0.2400	0.7246	0.6079	0
Masked Entropy (Cond)	0.7196	0.4294	0.3195	0.7125	0.3044	0.2308	0.7290	0.5976	0
Masked Entropy (Cond Span)	0.7429	0.4237	0.3477	0.7113	0.2408	0.2400	0.7855	0.6696	0
Entropy Gain (Cond)	0.3887	0.1401	0.0925	0.3849	0.1273	0.0608	0.3938	0.1572	0
Entropy Gain (Cond Span)	0.4855	0.1522	0.1301	0.4757	0.1473	0.0911	0.4988	0.1587	0
LGA Attribution	0.5945	0.3580	0.1977	0.5671	0.2650	0.1257	0.6314	0.4830	0
LDL Attribution	0.5839	0.3589	0.1967	0.5564	0.2607	0.1266	0.6208	0.4910	0
LGS Attribution	0.5051	0.1619	0.1400	0.5023	0.1340	0.0913	0.5088	0.1995	0
LIG Attribution	0.4995	0.1649	0.1355	0.4823	0.1306	0.0787	0.5227	0.2111	0
LGA Attribution (GO-mean)	0.6081	0.4050	0.2089	0.5768	0.3019	0.1304	0.6503	0.5438	0
LDL Attribution (GO-mean)	0.5961	0.4118	0.2099	0.5644	0.2922	0.1337	0.6389	0.5728	0
Attention Attribution	0.6167	0.3046	0.1608	0.5919	0.1908	0.0952	0.6501	0.4576	0
Naive	0.4958	0.1589	0.1196	0.4965	0.1408	0.0748	0.4950	0.1832	0
LGA Attribution (GO-max)	0.5894	0.4098	0.2017	0.5672	0.3133	0.1254	0.6193	0.5396	0
LGS Attribution (GO-mean)	0.5041	0.1660	0.1583	0.4913	0.1377	0.0948	0.5212	0.2040	0
Attention (layer 0)	0.5335	0.2365	0.1618	0.4972	0.1194	0.0950	0.5822	0.3941	0
LIG Attribution (GO-mean)	0.5219	0.1857	0.1526	0.5046	0.1688	0.0767	0.5452	0.2085	0
LIG Attribution (GO-sum)	0.5219	0.1857	0.1526	0.5046	0.1688	0.0767	0.5452	0.2085	0
LDL Attribution (GO-max)	0.5801	0.4068	0.2027	0.5552	0.3023	0.1273	0.6136	0.5474	0
Masked Entropy (Cond Span, 100/5)	0.7139	0.3560	0.3222	0.6683	0.1991	0.2258	0.7751	0.5671	0
Attention (layer 35)	0.4344	0.2426	0.1438	0.4158	0.1582	0.0905	0.4594	0.3560	0
LGS Attribution (GO-sum)	0.5041	0.1660	0.1583	0.4913	0.1377	0.0948	0.5212	0.2040	0
LC Attribution (GO-mean)	0.4980	0.1484	0.1528	0.4776	0.1125	0.0954	0.5253	0.1967	0
LIG Attribution (most-specific)	0.4995	0.1649	0.1355	0.4823	0.1306	0.0787	0.5227	0.2111	0
Masked Entropy (Cond Span, 200/5)	0.7248	0.3358	0.2709	0.7099	0.1923	0.1747	0.7448	0.5289	0
Attention (layer 9)	0.4859	0.3729	0.1329	0.5072	0.2924	0.0805	0.4573	0.4812	0
LC Attribution (most-specific)	0.4960	0.1488	0.1398	0.4723	0.1142	0.0850	0.5277	0.1954	0
Masked Entropy (Cond Span, 100/2)	0.7090	0.4292	0.3478	0.6537	0.2425	0.2439	0.7833	0.6803	0
LC Attribution (GO-max)	0.4912	0.1485	0.1407	0.4715	0.1065	0.0869	0.5177	0.2049	0
Masked Entropy (Cond Span, 50/5)	0.7216	0.4172	0.3421	0.6677	0.2300	0.2269	0.7942	0.6691	0
LGS Attribution (GO-max)	0.5072	0.1619	0.1532	0.4929	0.1196	0.0922	0.5265	0.2190	0
LGA Attribution (GO-sum)	0.6081	0.4050	0.2089	0.5768	0.3019	0.1304	0.6503	0.5438	0
LC Attribution (GO-sum)	0.4980	0.1484	0.1528	0.4776	0.1125	0.0954	0.5253	0.1967	0
Masked Entropy (Cond Span, 100/10)	0.7184	0.3155	0.3244	0.6791	0.1900	0.2263	0.7711	0.4844	0
LDL Attribution (GO-sum)	0.5961	0.4118	0.2099	0.5644	0.2922	0.1337	0.6389	0.5728	0
LIG Attribution (GO-max)	0.5237	0.1767	0.1558	0.5030	0.1584	0.0782	0.5515	0.2012	0
Attention (layer 18)	0.5847	0.3426	0.1678	0.5998	0.1945	0.0954	0.5645	0.5419	0
LDL Attribution (most-specific)	0.5839	0.3589	0.1967	0.5564	0.2607	0.1266	0.6208	0.4910	0
LGA Attribution (most-specific)	0.5945	0.3580	0.1977	0.5671	0.2650	0.1257	0.6314	0.4830	0
LGS Attribution (most-specific)	0.5043	0.1606	0.1421	0.4976	0.1432	0.0866	0.5134	0.1840	0

Table 24: Residue-level metrics on **Active Site**, stratified by the GO depth of `GOTerm[0]` (split at the dataset median). All proteins: n=465; Low depth: n=385; High depth: n=80. ΔAUC is high minus low.

**Method**	**All proteins**			**Low depth**			**High dept**		
	AUC	*MRR*	*Top-30*	*AUC*	*MRR*	*Top-30*	*AUC*	*MRR*	*T*
Masked Entropy	0.8235	0.5416	0.4540	0.8243	0.5130	0.4359	0.8194	0.6795	0
Masked Entropy (Cond)	0.8203	0.4671	0.4448	0.8226	0.5107	0.4305	0.8089	0.2574	0
Masked Entropy (Cond Span)	0.8368	0.4694	0.4577	0.8407	0.5027	0.4593	0.8184	0.3092	0
Entropy Gain (Cond)	0.3185	0.0740	0.0452	0.3250	0.0765	0.0453	0.2872	0.0619	0
Entropy Gain (Cond Span)	0.4859	0.1334	0.0976	0.4935	0.1425	0.1034	0.4497	0.0896	0
LGA Attribution	0.5763	0.1826	0.1729	0.5786	0.1702	0.1705	0.5656	0.2427	0
LDL Attribution	0.5697	0.2061	0.1718	0.5718	0.1976	0.1740	0.5592	0.2472	0
LGS Attribution	0.4929	0.0779	0.0796	0.4895	0.0738	0.0761	0.5094	0.0976	0
LIG Attribution	0.4763	0.1133	0.0885	0.4760	0.1241	0.0905	0.4777	0.0615	0
LGA Attribution (GO-mean)	0.5930	0.1988	0.1885	0.5995	0.1941	0.1910	0.5618	0.2216	0
LDL Attribution (GO-mean)	0.5802	0.2296	0.1912	0.5868	0.2361	0.1953	0.5487	0.1987	0
Attention Attribution	0.6716	0.3855	0.1999	0.6909	0.4127	0.2010	0.5786	0.2547	0
Naive	0.4990	0.1221	0.0969	0.4975	0.1224	0.0959	0.5057	0.1209	0
LGA Attribution (GO-max)	0.5805	0.1773	0.1693	0.5883	0.1635	0.1768	0.5428	0.2438	0
LGS Attribution (GO-mean)	0.4953	0.0768	0.0771	0.4991	0.0697	0.0778	0.4773	0.1110	0
Attention (layer 0)	0.5366	0.1461	0.1104	0.5311	0.1402	0.1101	0.5629	0.1745	0
LIG Attribution (GO-mean)	0.4748	0.1225	0.0970	0.4666	0.1275	0.0939	0.5146	0.0981	0
LIG Attribution (GO-sum)	0.4748	0.1225	0.0970	0.4666	0.1275	0.0939	0.5146	0.0981	0
LDL Attribution (GO-max)	0.5695	0.2000	0.1695	0.5775	0.1929	0.1794	0.5309	0.2342	0
Masked Entropy (Cond Span, 100/5)	0.8424	0.4741	0.4515	0.8412	0.5069	0.4505	0.8482	0.3167	0
Attention (layer 35)	0.3430	0.1810	0.0952	0.3388	0.1855	0.0907	0.3631	0.1595	0
LGS Attribution (GO-sum)	0.4953	0.0768	0.0771	0.4991	0.0697	0.0778	0.4773	0.1110	0
LC Attribution (GO-mean)	0.4604	0.0665	0.0765	0.4619	0.0677	0.0790	0.4534	0.0608	0
LIG Attribution (most-specific)	0.4763	0.1133	0.0885	0.4760	0.1241	0.0905	0.4777	0.0615	0
Masked Entropy (Cond Span, 200/5)	0.7952	0.3878	0.3496	0.8047	0.4244	0.3577	0.7498	0.2115	0
Attention (layer 9)	0.5255	0.2603	0.1109	0.5319	0.3088	0.1298	0.4948	0.0265	0
LC Attribution (most-specific)	0.4614	0.0674	0.0753	0.4624	0.0703	0.0779	0.4563	0.0538	0
Masked Entropy (Cond Span, 100/2)	0.8571	0.5007	0.4835	0.8569	0.5334	0.4812	0.8580	0.3432	0
LC Attribution (GO-max)	0.4632	0.0775	0.0791	0.4634	0.0794	0.0819	0.4622	0.0684	0
Masked Entropy (Cond Span, 50/5)	0.8333	0.4299	0.4503	0.8437	0.4287	0.4708	0.7835	0.4356	0
LGS Attribution (GO-max)	0.4949	0.0785	0.0819	0.4969	0.0774	0.0814	0.4852	0.0839	0
LGA Attribution (GO-sum)	0.5930	0.1988	0.1885	0.5995	0.1941	0.1910	0.5618	0.2216	0
LC Attribution (GO-sum)	0.4604	0.0665	0.0765	0.4619	0.0677	0.0790	0.4534	0.0608	0
Masked Entropy (Cond Span, 100/10)	0.8157	0.3242	0.3950	0.8098	0.3322	0.3774	0.8438	0.2858	0
LDL Attribution (GO-sum)	0.5802	0.2296	0.1912	0.5868	0.2361	0.1953	0.5487	0.1987	0
LIG Attribution (GO-max)	0.4775	0.1219	0.1034	0.4708	0.1255	0.1012	0.5101	0.1047	0
Attention (layer 18)	0.6635	0.3479	0.2062	0.6498	0.3443	0.1720	0.7296	0.3651	0
LDL Attribution (most-specific)	0.5697	0.2061	0.1718	0.5718	0.1976	0.1740	0.5592	0.2472	0
LGA Attribution (most-specific)	0.5763	0.1826	0.1729	0.5786	0.1702	0.1705	0.5656	0.2427	0
LGS Attribution (most-specific)	0.4859	0.0756	0.0778	0.4859	0.0786	0.0813	0.4863	0.0608	0

Table 25: Residue-level metrics on **Homologous Superfamily**, stratified by the GO depth of `GOTerm[0]` (split at the dataset median). All proteins: n=257; Low depth: n=164; High depth: n=93. ΔAUC is high minus low.

**Method**	**All proteins**			**Low depth**			**High depth**		
	AUC	*MRR*	*Top-30*	*AUC*	*MRR*	*Top-30*	*AUC*	*MRR*	*Top-30*
Masked Entropy	0.4141	0.3751	0.2184	0.3815	0.1503	0.1443	0.4716	0.7715	0.3491
Masked Entropy (Cond)	0.4229	0.3015	0.2316	0.3987	0.1670	0.1648	0.4655	0.5386	0.3495
Masked Entropy (Cond Span)	0.3560	0.3297	0.1988	0.3336	0.2006	0.1055	0.3955	0.5574	0.3634
Entropy Gain (Cond)	0.5229	0.4933	0.3185	0.5162	0.4240	0.2488	0.5346	0.6154	0.4416
Entropy Gain (Cond Span)	0.4933	0.3946	0.2601	0.4813	0.3257	0.1976	0.5145	0.5161	0.3703
LGA Attribution	0.4565	0.4751	0.2486	0.4681	0.5233	0.2246	0.4361	0.3902	0.2910
LDL Attribution	0.4651	0.4676	0.2549	0.4767	0.5217	0.2268	0.4446	0.3721	0.3043
LGS Attribution	0.4702	0.2731	0.2358	0.4844	0.2468	0.2083	0.4452	0.3195	0.2842
LIG Attribution	0.4861	0.4992	0.2750	0.4731	0.4552	0.2289	0.5091	0.5767	0.3563
LGA Attribution (GO-mean)	0.4539	0.4332	0.2332	0.4776	0.4444	0.2173	0.4121	0.4134	0.2613
LDL Attribution (GO-mean)	0.4662	0.4319	0.2348	0.4847	0.4545	0.2150	0.4336	0.3920	0.2695
Attention Attribution	0.5202	0.5383	0.3052	0.5027	0.5139	0.2364	0.5510	0.5813	0.4265
Naive	0.5035	0.4972	0.3171	0.5040	0.4546	0.2677	0.5025	0.5724	0.4043
LGA Attribution (GO-max)	0.4573	0.4287	0.2414	0.4767	0.4563	0.2157	0.4231	0.3802	0.2867
LGS Attribution (GO-mean)	0.4790	0.2508	0.2479	0.5001	0.2249	0.2327	0.4419	0.2965	0.2746
Attention (layer 0)	0.5030	0.5859	0.2502	0.4795	0.7039	0.2362	0.5444	0.3778	0.2749
LIG Attribution (GO-mean)	0.4605	0.4672	0.2686	0.4516	0.4468	0.2091	0.4761	0.5032	0.3735
LIG Attribution (GO-sum)	0.4605	0.4672	0.2686	0.4516	0.4468	0.2091	0.4761	0.5032	0.3735
LDL Attribution (GO-max)	0.4694	0.4374	0.2396	0.4847	0.4634	0.2096	0.4424	0.3915	0.2925
Attention (layer 35)	0.4989	0.4124	0.2674	0.4770	0.3181	0.1951	0.5374	0.5788	0.3950
LGS Attribution (GO-sum)	0.4790	0.2508	0.2479	0.5001	0.2249	0.2327	0.4419	0.2965	0.2746
LC Attribution (GO-mean)	0.4971	0.3390	0.2514	0.4981	0.3062	0.2169	0.4954	0.3968	0.3122
LIG Attribution (most-specific)	0.4861	0.4992	0.2750	0.4731	0.4552	0.2289	0.5091	0.5767	0.3563
Attention (layer 9)	0.5076	0.3376	0.3489	0.4796	0.2807	0.2569	0.5570	0.4379	0.5111
LC Attribution (most-specific)	0.4952	0.3454	0.2569	0.4941	0.3075	0.2195	0.4972	0.4121	0.3229
LC Attribution (GO-max)	0.4971	0.3650	0.2581	0.4903	0.3217	0.2067	0.5092	0.4413	0.3487
LGS Attribution (GO-max)	0.4857	0.2974	0.2747	0.4979	0.2701	0.2451	0.4642	0.3455	0.3269
LGA Attribution (GO-sum)	0.4539	0.4332	0.2332	0.4776	0.4444	0.2173	0.4121	0.4134	0.2613
LC Attribution (GO-sum)	0.4971	0.3390	0.2514	0.4981	0.3062	0.2169	0.4954	0.3968	0.3122
LDL Attribution (GO-sum)	0.4662	0.4319	0.2348	0.4847	0.4545	0.2150	0.4336	0.3920	0.2695
LIG Attribution (GO-max)	0.4650	0.4666	0.2857	0.4574	0.4397	0.2278	0.4783	0.5141	0.3878
Attention (layer 18)	0.4963	0.5494	0.3137	0.4909	0.5003	0.2787	0.5059	0.6361	0.3756
LDL Attribution (most-specific)	0.4651	0.4676	0.2549	0.4767	0.5217	0.2268	0.4446	0.3721	0.3043
LGA Attribution (most-specific)	0.4565	0.4751	0.2486	0.4681	0.5233	0.2246	0.4361	0.3902	0.2910
LGS Attribution (most-specific)	0.4723	0.2859	0.2451	0.4800	0.2461	0.2091	0.4587	0.3559	0.3086
